# Multi‐environment evaluation and genomic prediction of agronomic traits in the southern US rice genepool

**DOI:** 10.1002/tpg2.70222

**Published:** 2026-03-30

**Authors:** Mary‐Francis LaPorte, Haixiao Hu, Walter Solomon, James H. Oard, Steven J. Knapp, Daniel E. Runcie, Jeremy D. Edwards, Anna McClung, Christine H. Diepenbrock

**Affiliations:** ^1^ Department of Plant Sciences University of California Davis California USA; ^2^ Delta Research and Extension Center Mississippi State University Stoneville Mississippi USA; ^3^ Department of Agronomy, LSU AgCenter Louisiana State University Baton Rouge Louisiana USA; ^4^ USDA‐ARS Dale Bumpers National Rice Research Center Stuttgart Arkansas USA

## Abstract

The southern United States is responsible for 80% of the country's production of rice, approximately half of which is exported. Understanding genotypic and environmental factors impacting the historical performance of rice (*Oryza sativa* L.) is important for directing research efforts to optimize production of this globally important crop. A set of 429 rice genotypes including globally diverse historical parents and advanced *japonica* breeding lines from southern US breeding programs was phenotyped in 2008 for eight agronomic traits in Arkansas, Louisiana, and Mississippi. They were also genotyped using a single‐nucleotide polymorphism set optimized for genomic prediction/selection. Genotypic and phenotypic data were analyzed via clustering techniques and principal component analysis. Single‐trait and multi‐trait genomic prediction were used to predict genetic values on a per‐plant basis. We found that contemporary germplasm from the southern state breeding programs was highly interrelated and distinct from progenitor *indica* and temperate *japonica* genotypes. Genomic predictive abilities were high and largely consistent across environments for seed number per panicle, tiller number, and plant height. Although predictive abilities were lower for seed weight per panicle, that trait was correlated with seed number per panicle (*r* = 0.919), and predictive ability was higher for both traits in a multi‐trait prediction framework. Furthermore, including data from the two major genotypic clusters identified herein had no penalty on genomic predictive ability. The data and analyses presented herein could inform future genomic and phenotypic investigations and applied breeding in the southern US rice germplasm pool.

## Core Ideas

- We present a diverse, 429‐genotype rice collection, which was phenotyped in three states in the Mississippi Delta.
- Analyses of the population structure highlighted breeding program origins, and principal component (PC) plots evidenced admixture.
- Multi‐trait genomic prediction generally conferred a predictive advantage over the already‐high genomic best linear unbiased prediction (GBLUP) predictive abilities.

## Plain Language Summary

Genetics and growing environment impact how a rice plant grows. We studied the impacts of genetics and environment on a set of 429 rice genotypes by measuring differences in growth traits when the rice was grown in three states in the southern United States. These traits include the height of the plant, the number of seed‐bearing shoots, and the total seed weight, along with flowering time and maturity time. We characterized the genetic diversity of the rice genotypes and predicted breeding values for the measured traits in two different ways: (1) using only genetic information and (2) using genetic information combined with measurements of other traits. We found method (2) had the highest accuracy. We also found that method (1) performed similarly in each environment, indicating that the dataset and resources provided in this study could help support future prediction/selection efforts in rice.

**Abbreviations**
GBLUP: genomic best linear unbiased prediction
GP: genomic prediction
G × E: genotype‐by‐environment
MegaLMM: mega‐scale linear mixed models
PC: principal component
PCA: principal component analysis
RiceCAP: Rice Coordinated Agricultural Project
RR‐BLUP: ridge regression best linear unbiased prediction
SNP: single‐nucleotide polymorphism
TASSEL: Trait Analysis by aSSociation, Evolution and Linkage
URRN: uniform regional rice nursery

## 1 INTRODUCTION

The United States is the 13th largest producer of rice (*Oryza sativa* L.) globally and is regularly in the top five rice‐exporting nations (Childs & Jarrell, 2024; Wang et al., 2024). About one‐third of the US crop is exported (33.4% in October 2024), and Arkansas has the largest production and area harvested of rice by state within the US (Childs & Jarrell, 2024; US Department of Agriculture Foreign Agricultural Service, 2023). Rice produced in the southern US is primarily derived from genotypes introduced in the late 1800s and early 1900s by way of southeast Asia, Japan, Madagascar, Italy, and Central America (Dilday, 1990; Rutger & Bollich, 1991). Twenty‐two historical introductions heavily influenced the germplasm of the region, primarily ssp. *japonica* varieties, which gave rise to modern genotypes (Dilday, 1990). The progeny of crosses among these genotypes were bred for various cooking quality, aroma, grain amylose content, grain sizes, and yield (Angira et al., 2022; Ayres et al., 1997; Glaszmann, 1987; Lu et al., 2005; Rutger & Bollich, 1991). To discover more regarding the population structure of the germplasm and to aid breeding efforts, Lu et al. (2005) genotyped, phenotyped, and analyzed the diversity of 145 genotypes relevant to the southern US. Results from that study and others demonstrated that although there has been some introduction of specific genes and traits as evidenced by admixture, the population structure of the US rice genepool has largely remained unchanged since its establishment (Lu et al., 2005; Vaughn et al., 2021; Wang et al., 2024), indicating adequate genetic variation exists within the *japonicas* for long‐term breeding. More recently, Wang et al. (2024) indicated that utilization of temperate × tropical *japonica* intercrosses may support greater resilience to predicted future climate changes in the southern US.

Genomic selection (GS) uses predictive models trained on genomic markers as a support tool for breeding decisions to drive genetic gain (Meuwissen, 2009; Meuwissen et al., 2001). GS has become an increasingly important strategy for improving rice, including rice adapted to the southern US (da Silva et al., 2025; Nguyen et al., 2023; Spindel & Iwata, 2018; Xu et al., 2021). Genomic prediction (GP) models, which are the statistical models central to GS, predict breeding values for a phenotype of interest using genomic markers as predictors; for example, genomic best linear unbiased prediction (GBLUP) is a classical method used in GP/GS (Clark & Werf, 2013; Crossa et al., 2022; Meuwissen, 2009; Meuwissen et al., 2001). A genotyping panel was recently created for efficient and cost‐effective genotyping at single‐nucleotide polymorphisms (SNPs) that capture genetic variation within and across southern US rice breeding programs (Cerioli et al., 2022), which is critical for routine implementation of GP/GS. In addition, GP is designed to capture smaller effects across the genome rather than relying solely on one or a couple of major‐effect loci, which can be helpful for genetically complex traits (Crossa et al., 2017; Xu et al., 2021). This rapid genotyping method enables selection at the seedling stage, allowing rapid identification of progeny for further crossing or advancement that can ultimately increase the efficiency and effectiveness of the breeding programs. A limitation of classical GP is that it only incorporates genotypic data to predict breeding values for a given trait. One way to address this limitation is to use “secondary” traits (i.e., traits other than the trait of interest that are biologically relevant and/or easily measured in the same or similar environment(s), or the same trait measured in a different environment) as predictors, as has been implemented in methods such as MegaLMM (mega‐scale linear mixed models) for GP (Runcie et al., 2021). The relationships between the trait of interest and the same or secondary traits in other environments can improve predictive ability. Additionally, intermediate traits that are measured in the same environment as the trait of interest could predict difficult, time‐consuming, expensive‐to‐measure, and/or late‐stage traits in the same environment (Hu et al., 2024; Runcie et al., 2021).

In 2004, a cooperative multistate project involving US rice geneticists and breeders (USDA Rice Coordinated Agricultural Project [RiceCAP]) was created to develop genomic tools to select for agronomic (including phenological, architectural, and yield‐component) traits, sheath blight resistance, and milling quality within the southern US rice genepool. For this purpose, a core set of 429 genotypes was assembled and grown, including released genotypes and contemporary advanced breeding lines developed from the southern public breeding programs located in Arkansas, Louisiana, Mississippi, and Texas, along with historical genotypes from the California breeding program and from other countries that had been used as parent material. Through over a century of rice breeding in the US, tropical *japonica* germplasm had been found to be better adapted to the southern US, whereas temperate *japonicas* were best suited to the California climate. This environmental adaptation and the difference in quality expected from the temperate medium grain and the tropical long grain market classes sustained the distinct genepools used in these two regions. The set of 429 genotypes was evaluated in three southern environments in 2008 to better understand phenotypic performance and its variance and covariance across genotypes in the southern US.

In the present study, we conducted genotypic and multi‐environment phenotypic analyses and single‐ and multi‐trait GP using a panel of 429 genotypes that was field‐evaluated at one research station in each of Louisiana, Mississippi, and Arkansas. By using various analyses of genotypic and phenotypic data, and single‐ and multi‐trait GP approaches, we explore the genotypic and environmental basis of these complex traits and report on this unique dataset and germplasm collection. The analyses conducted herein could assist in strategizing cost‐effective phenotyping approaches within and across southern US environments.

**Core Ideas**

- We present a diverse, 429‐genotype rice collection, which was phenotyped in three states in the Mississippi Delta.
- Analyses of the population structure highlighted breeding program origins, and principal component (PC) plots evidenced admixture.
- Multi‐trait genomic prediction generally conferred a predictive advantage over the already‐high genomic best linear unbiased prediction (GBLUP) predictive abilities.

## 2 MATERIALS AND METHODS

### 2.1 Population and phenotypic data

The 429 genotypes phenotyped in this study included 84 historical genotypes released from the southern US breeding programs, 22 genotypes released from the California breeding program, 27 introductions from other countries used as breeding parents, and 301 advanced breeding genotypes from the southern Uniform Regional Rice Nursery (URRN) (from the 2000–2006 time period), with some genotypes falling in multiple categories (Table S1). The seed was purified by single‐seed descent and increased for planting in 2007. The genotypes were grown in 2008 at the H. Rouse Caffey Rice Research Station in Crowley, LA; the Delta Research Extension Center in Stoneville, MS; and the Dale Bumpers National Rice Research Center in Stuttgart, AR (Figure S1). The field study consisted of two replications in a randomized complete block design with plots planted as hill drops in a single row, 5 m in length, with a spacing of 0.25 m between the plants and 0.50 m between the rows. Prior to planting, phosphorus and potassium were incorporated according to local recommendations. Approximately 30 days after seeding, plots were flooded, and urea nitrogen was applied at a reduced rate of 56 kg ha^−1^ due to the low seeding rate. Experiments were maintained under flood throughout the season. Phenotypic measurements were determined on two to three plants per plot in all environments and included heading date, maturity date, days to grainfill, plant height, tiller number, panicle length, seed number per panicle, and seed weight per panicle. In Mississippi and Arkansas, total seed weight was also measured for all genotypes, and total aboveground biomass was measured for 191 and 174 genotypes, respectively. A Köppen–Geiger map of the location of these research sites was created using QGIS and state boundaries as defined by the US Census Bureau (Figure S1; Beck et al., 2023; Dawson et al., 2025; US Census Bureau, 2023).

### 2.2 Genotypic data

In 2020, these genotypes were grown in a greenhouse in Stuttgart, AR. Leaf disks were collected and sent to Agriplex Genomics for genotyping using the Agriplex 550 SNP Rice panel designed for GS using southern US rice germplasm (Cerioli et al., 2022). In brief, this SNP panel was derived initially from a set of 1200 polymorphic SNPs deemed informative for southern US rice and well‐distributed throughout the genome, originating from the C7AIR rice SNP array (7K) (Morales et al., 2020). From these 1200 polymorphic SNPs, 550 SNPs were selected and became known as the LSU500 panel. The distribution of the LSU500 SNPs is reported in Cerioli et al. (2022). 549 of the 550 SNPs were polymorphic in the germplasm under study herein. No sites were missing, but only 491 were included after filtering for a minor allele frequency of 0.05. The final number of SNPs used for prediction was 491.

### 2.3 Genotypic and phenotypic analyses

The 550 SNP dataset was analyzed via principal component analysis (PCA). The eigenvector/eigenvalue pairs were calculated using the PCA function in the Trait Analysis by aSSociation, Evolution and Linkage (TASSEL 5) software (Bradbury et al., 2007) using genotypic data transformed to numeric with the homozygous major allele set to 1, homozygous minor set to 0, and heterozygotes set to 0.5. The results were visualized using ggplot2 (Wickham, 2016) in R (R Core Team, 2021). *K*‐means clustering was conducted on PC1 and PC2 (where PC is Principal Component) using the kmeans() function with the argument *k* = 2, for purposes of having a sufficient sample size within a subgroup to conduct GP. This clustering resulted in a set of genotypes referred to as Group A and Group B. Annotations for the breeding program of record for the different genotypes were manually curated using the URRN records and crop registration articles (Table S1).

The STRUCTURE and ADMIXTURE softwares for genetic inference (Alexander et al., 2009; Pritchard et al., 2000) were used to infer population structure based on SNP data. The STRUCTURE software was run with a burn‐in length of 10 and 50 replications of Markov chain Monte Carlo after burn‐in. This process was repeated with all values of *k* = 1 through *k* = 10. The ADMIXTURE software was run for values of *k* = 1 through *k* = 30. No clear value for *k* could be determined from the results (Figure S2). A genomic heatmap was created using the kinship matrix, created using the TASSEL 5 software (Bradbury et al., 2007) kinship function, and the heatmap() function from R (Figure S3). The manual annotations were those reported in this study, and ADMIXTURE cross‐validation error is reported in the supplement.

The eigenvector/eigenvalue pairs were calculated for the phenotypic PCA plots using the prcomp() function in base R with scale set to true, and the fviz_eig() function from the factoextra package (Kassambara & Mundt, 2016). These components were calculated twice: once using the average of each trait over all three environments, and again with each trait separated by the environment in which it was collected. The results were visualized using the fviz_pca_biplot() function from factoextra using default settings. The correlogram was generated using the ggpairs() function from ggplot2 on the phenotypic data using default settings (Wickham, 2016).

The standard deviation relative to the mean, or coefficient of variation, was calculated by dividing the standard deviation by the mean and multiplying by 100. This calculation was conducted for each trait, both cumulatively for all environments and within each environment. Genotype‐by‐environment (GxE) analysis was conducted using the createVarComp() function and a custom modification of the vc() function from the statgenGxE package (Rossum, 2024). The modified script allowed for the linear models to better represent the experimental design, with trait as the response and random effects for environment, genotype, replicate nested in environment, and the genotype‐environment interaction term. A plotting function for the output figures was also adapted.

The GBLUP method was used for single‐trait GP using the TASSEL 5 GS plugin (Bradbury et al., 2007; LaPorte et al., 2024). A kinship matrix was created from the SNP dataset using the TASSEL kinship matrix function. GBLUP was conducted using 20 iterations of fivefold cross‐validation for each trait in each environment with random assignment of genotypes to folds, a typical framework for GP. GBLUP was conducted both in the complete set of genotypes and in genotypes solely from Group A (determined via *k*‐means clustering, as described above).

Multi‐trait GP was conducted using MegaLMM (Runcie, 2025; Runcie et al., 2021) using 100 iterations of twofold cross‐validation with random assignment of genotypes to folds. Twofold cross‐validation allowed for sufficient genotypes to be in the training and test set to perform MegaLMM. We used a single trait from a given environment, averaged over two replications per genotype, as the focal trait. The MegaLMM model was trained on half of the values from that focal trait in the focal environment plus all data from all traits in the other two environments, with the remaining half of the observations of the focal trait in the focal environment masked. The total genetic value (*Y*) and estimated additive genetic value (*U*) were reported from MegaLMM. *Y* represents the expected phenotype for each genotype, while *U* is the estimated portion of that genetic value that is heritable from the additive effects of individual genes. The *Y* value of the focal trait (trait of interest) was then predicted from this model—representing the total genetic value predicted for a particular genotype for the focal trait in the focal environment. Predictive ability was calculated as the correlation between this predicted *Y* value and the observed phenotypic value (averaged across two replicates per genotype in that environment) of the test set, withheld during model training.

MegaLMM was also used to predict the additive genetic value (*U*) and estimate model accuracy, as the correlation between the predicted and true additive genetic value, using the estimate_gcor() function (Runcie et al., 2021). A single‐trait prediction for a given trait, using ridge regression‐best linear unbiased prediction (RR‐BLUP) via mixed.solve() from the rrBLUP package, was also calculated using the function mixed.solve() from the rrBLUP package (Endelman, 2011) using 100 iterations of twofold cross‐validation for direct comparison with multi‐trait prediction. The mixed.solve() implementation from rrBLUP is mathematically equivalent to the GBLUP implementation from TASSEL. This direct comparison of MegaLMM and RR‐BLUP was conducted using the exact same fold assignments for multi‐trait and single‐trait GP. Total seed weight was not predicted for Louisiana as it was not measured in that environment. Total biomass was not predicted in any environment because values were missing for too many genotypes, even for the environments in which that trait was partially measured (Arkansas and Mississippi). The cov2cor() function was used to calculate genetic correlations from the posterior genetic covariance matrix created by MegaLMM.

## 3 RESULTS AND DISCUSSION

### 3.1 Panel origin

We describe a rice germplasm panel, including historical and contemporary genotypes, that was assembled by the US rice breeding community to explore genotype and environment interactions in the predominant rice growing region of the Mississippi Delta. As rice is not indigenous to the US, the establishment of the domestic rice industry was initiated by the introduction of varieties primarily from various parts of Asia, but also Central America, Madagascar, and Europe (Dilday, 1990). These progenitor varieties were the core materials for US breeding programs which became a relatively narrow, predominantly tropical *japonica*, genepool in the Mississippi Delta growing region. This study evaluated 429 genotypes that included diverse parental varieties as well as elite breeding lines from breeding programs located in Arkansas, Louisiana, Mississippi, and Texas.

**FIGURE 1:**
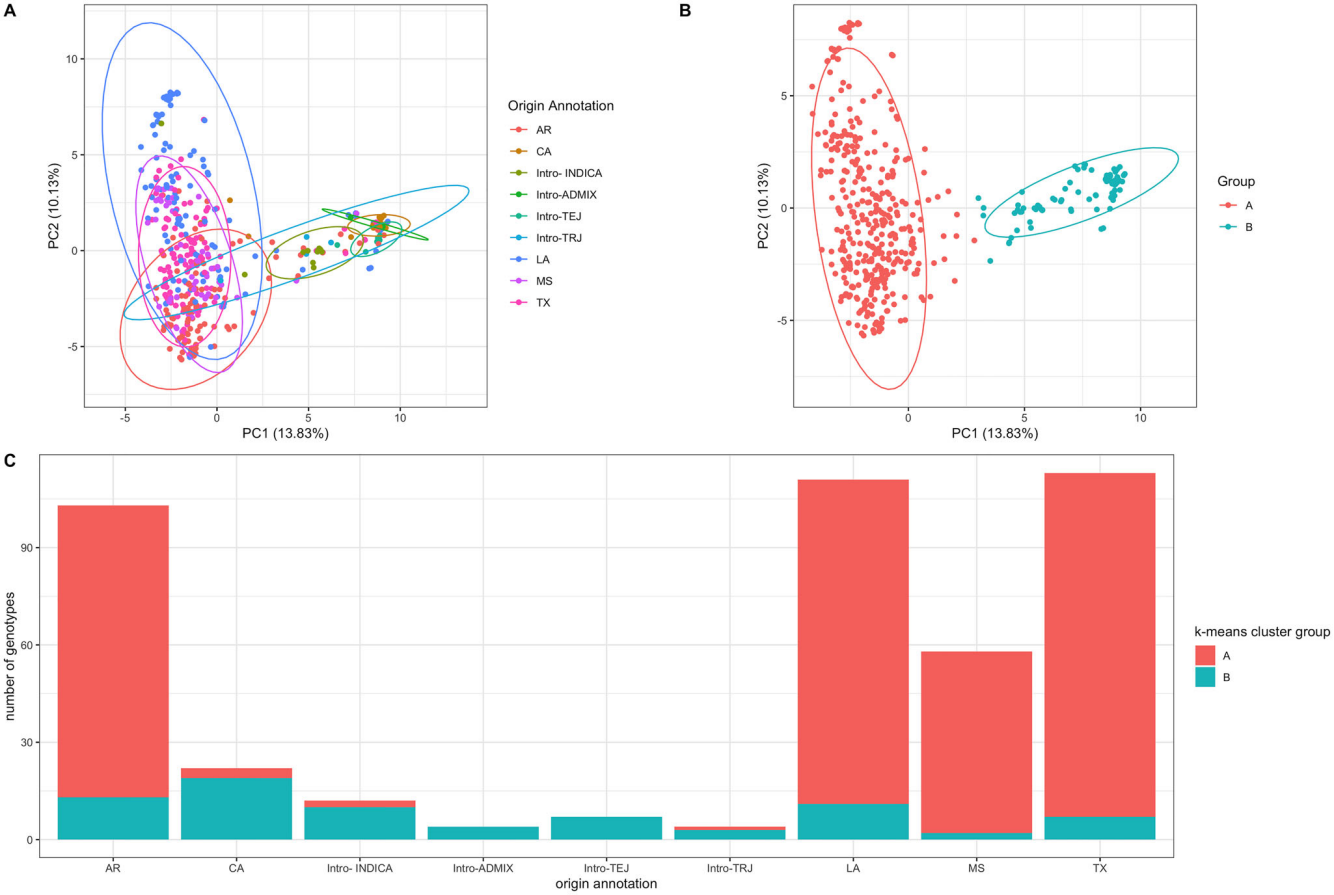
Principal component analysis (PCA) of genotypic data, and summary of *k*‐means group membership by annotated origin. AR: Arkansas, CA: California, LA: Louisiana, MS: Mississippi, and TX: Texas are state abbreviations, indicating that a breeding program in that state developed a given genotype. “INDICA” refers to an *indica* genotype originating from outside of the listed breeding programs. “NA” (not available) signifies that no origin annotation was available for a particular genotype. Lines denote normal data ellipses (stat_ellipse()). The percent variance explained by each principal component (PC) is reported in parentheses in the respective axis label. Panel (A): PCA of genotypic data showing PC1 versus PC2, with rice genotypes color‐coded based on annotated origin. (B) Genotypic PCA showing PC1 versus PC2, with rice genotypes color‐coded based on *k*‐means clustering (*k* = 2), labeled as Group A and Group B. (C) Number of genotypes in each annotated origin group attributed to each *k*‐means group (A and B).

**FIGURE 2:**
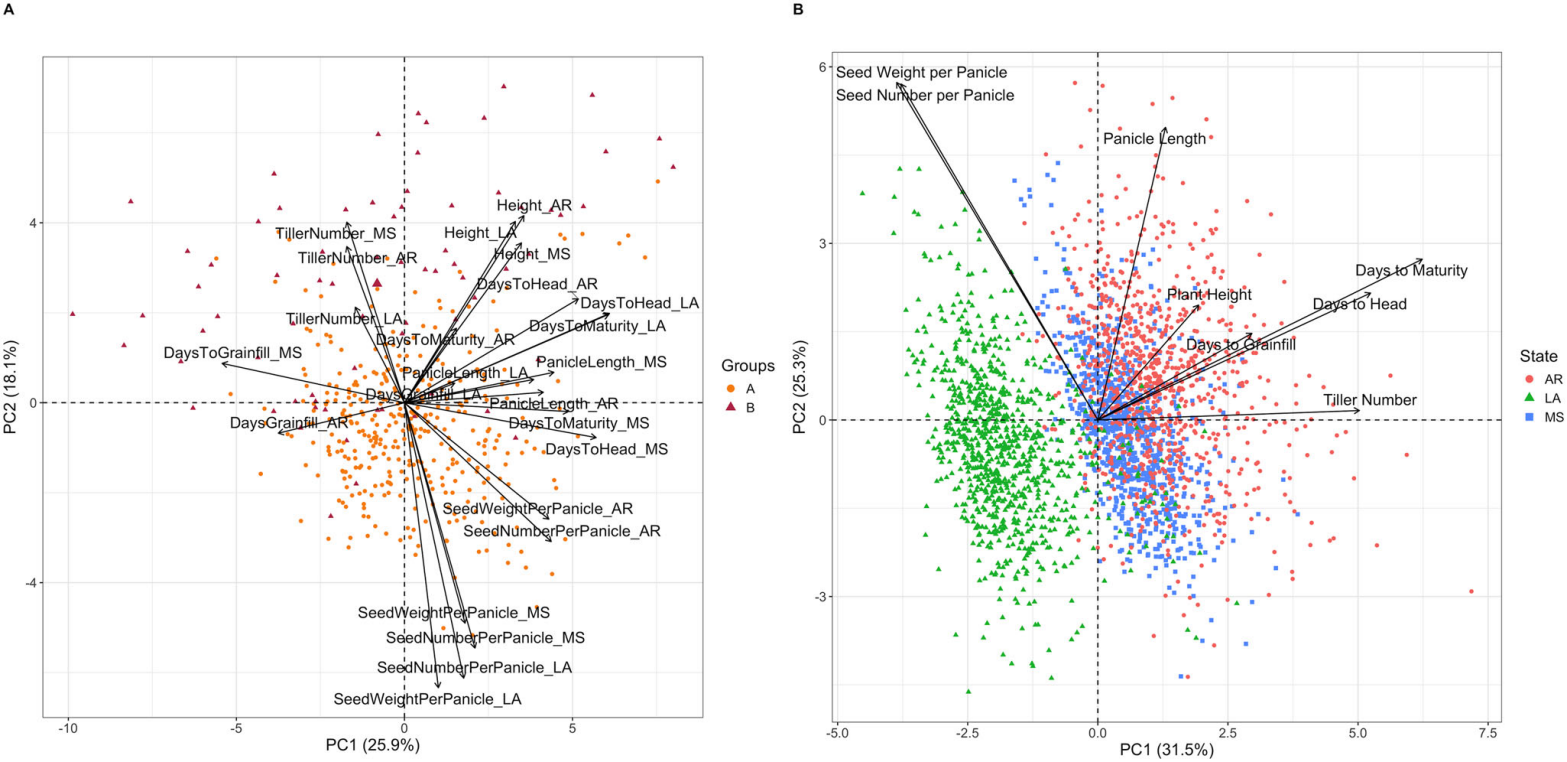
(A) Principal component analysis (PCA) of phenotypic data. The color and shape of the points represent the group to which a genotype was assigned through *k*‐means clustering based on genotypic data. Vectors represent contributing variables for the principal component (PC) dimensions, which for this plot were specific to the environment in which each phenotype was measured (i.e., one vector for each trait‐environment combination; AR: Arkansas; LA: Louisiana; or MS: Mississippi). The percent variance explained by each PC is reported in parentheses in the respective axis label. (B) PC analysis of phenotypic data. The color and shape of the points represent the state in which phenotypic data were collected (AR: Arkansas; LA: Louisiana; or MS: Mississippi). Vectors represent contributing variables for the PC dimensions, which for this plot were the average trait value across all three environments (i.e., one vector for each trait). The percent variance explained by each PC is reported in parentheses in the respective axis label.

**FIGURE 3:**
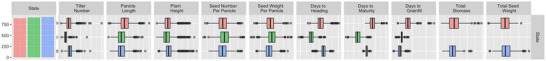
Boxplots for phenotypes measured in each environment. The colors represent the state in which phenotypes were measured (AR: Arkansas in red; LA: Louisiana in green; MS: Mississippi in blue). For most traits and most environments 429 genotypes were measured (apart from total biomass, which was only measured for 157 genotypes in Arkansas and 198 genotypes in Mississippi). The total number of genotypes observed in each environment is summarized in the bar chart to the far left titled “State.”

Based on genotypic data, the rice accessions separated in PC space (Figure 1A,B). Two major sub‐groupings were created based on the genotypic data using *k*‐means clustering: Groups A and B contained 375 and 81 genotypes, respectively (summing to 456 total, the number of genotypes that had been genotyped for this study, which contains the set of 429 lines that were phenotyped; Figure 1B). The two groups separated along PC1, which explained 13.8% of the variance. Genotypes intermediate between the two groups indicated potential shared genetic regions (Figures 1 and 2). Annotations of the origins of these rice genotypes included southern rice breeding programs (abbreviated by the state: Arkansas, Louisiana, Mississippi, and Texas), the California breeding program, and foreign introductions (Figure 1A). A majority of the rice accessions annotated as belonging to a southern US breeding program (AR, LA, MS, and TX) were members of Group A, with smaller proportions belonging to Group B (Figure 1C; Table S1). The region of the genotypic PC plot containing Group A (Figure 1B) contained the centroid of the genotypes annotated with Louisiana, Mississippi, Texas, and Arkansas (Figure 1A); the genotypes with the highest values on the PC2 axis came from Louisiana. A majority of the accessions annotated as belonging to the CA breeding program, temperate or tropical *japonica* introduction (TEJ or TRJ, respectively), or an *indica* or admixture introduction were members of Group B (Figure 1C; Table S1), which contained several medium‐grain genotypes derived from temperate *japonica* crosses (Table S1; Figure 1A,C; Figure 3). The intermediate genotypes between Groups A and B contained the majority of the *indica* genotypes, which were grouped together, as well as genotypes from each breeding program (Figure 1A). From the kinship heatmap analysis, the varieties that included TEQING, a medium‐grain *indica* introduction, formed a correlated cluster (Figure S3).

Although this rice population partially clustered by breeding program in PC space (most notably for Louisiana and California), there were many overlaps (Figure 1A), which is likely due to the common use of historic founder lines and the interbreeding among subsequent releases from the different breeding programs that were targeting distinct environments. Dilday (1990) studied the interrelatedness of rice varieties from major US rice breeding programs and found that a limited number of genotypes contributed the genetics for each program. Genotypes characterized by Dilday overlapped with those included in this analysis. However, the pedigree of a specific cross does not alone determine the genetic variability that is utilized in a breeding program. The breeding strategy, including the number of offspring evaluated, selection timing, and trait priorities, will also determine the genetic potential for variety development. Lu et al. (2005) used simple sequence repeats to characterize the genetic diversity of 145 US rice genotypes. That study described separation by market class rather than breeding program and identified four categories: temperate *japonica* short/medium grain, tropical *japonica* medium grain, tropical *japonica* long grain, and *indica*. The present study contained over twice as many genotypes as those described in Dilday (1990) and Lu et al. (2005) and separated them into two clusters using a genotypic PCA as described above.

### 3.2 Phenotypic analyses

The traits analyzed in this study included heading date, maturity date, days to grainfill, plant height, tiller number, panicle length, seed number per panicle, and seed weight per panicle in all three environments, as well as total biomass and total seed weight in Arkansas and Mississippi only. The first PC of the phenotypic data (averaged across the three environments) aligned most closely with the growing environment in which the genotype was phenotyped in this study and explained 31.5% of the variance (Figure 2B). The second PC, however, had a similar variance distribution for each of the three environments (with the genotypes in the population spread along PC2 in all three environments) and explained 25.3% of the variance. Several genotypes annotated with *indica* origin had among the highest values for tiller number in every environment, which is consistent with previous findings that *indica* genotypes tend to have higher tiller numbers than non‐*indica* genotypes (Liu et al., 2024; Ma et al., 2020; Ren et al., 2021).

A PCA on phenotypic traits across genotype:environment combinations revealed separation of the environments along PC1 and PC2, which respectively explained 25.9% and 18.1% of the variance (Figure 2A). Louisiana was a somewhat distinct environment from Arkansas and Mississippi, which were largely similar and overlapping. For both PCs, the lowest values were in the Louisiana environment, while the highest values were in Arkansas. The axis of separation between the environments was most closely related to PC1. The phenological traits (days to maturity, days to heading, and days to grainfill) were correlated with each other but differed between environments (Figure 2A,B). Seed number per panicle and seed weight per panicle were highly correlated within environments (Figure 2A; Figures S4 and S5).

The separation of Groups A and B, identified using genotypic data, was also observed based on phenotypes (Figure 2A). The groups separated along PC2, and Group B was more spread along PC1 whereas Group A was more closely clustered around the origin. Group A had significantly higher seed weight per panicle (LA: *p* < 0.0001; MS: *p* < 0.001; AR: *p* < 0.05; Welch's *T*‐test) and seed number per panicle (*p* < 0.0001 in all states; Figure 2A; Table S1), whereas Group B had significantly higher tiller number in all environments (*p* < 0.0001; Welch's *T*‐test; Figure 2A; Table S1). In general, the trait vectors for a single given trait in each environment tended to group together, meaning the traits were correlated across environments (Figure 2A). Exceptions to this tendency included days to grainfill for Mississippi and Arkansas, which did not group with Louisiana. Additionally, days to heading and days to maturity grouped by state rather than by trait. Seed number per panicle and seed weight per panicle in Mississippi and Louisiana all grouped together, but those two traits grouped separately in Arkansas.

### 3.3 Single‐trait GP

**FIGURE 4:**
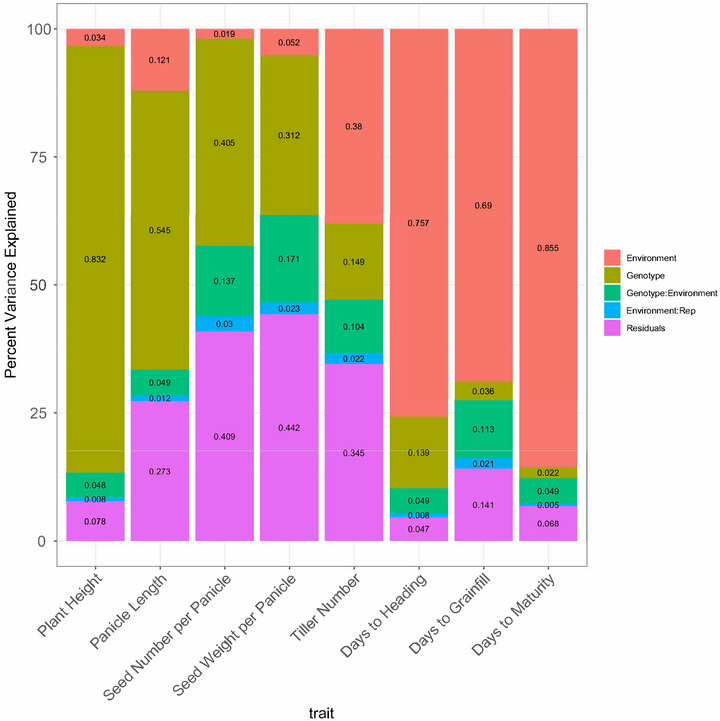
Percent variance explained by each predictor in a genotype‐by‐environment (G × E) analysis for the traits measured in all environments. The linear model represented in this figure is: Trait ∼ (1 | E) + (1 | G) + (1| E:Rep) + (1 | G:E) + Residual, where (1 | E) represents the random effect of environment, (1 | G) represents the random effect of genotype, (1| E:Rep) represents the random effect of replicate nested in environment, and (1 | G:E) represents the random effect of the G × E interaction.

**FIGURE 5:**
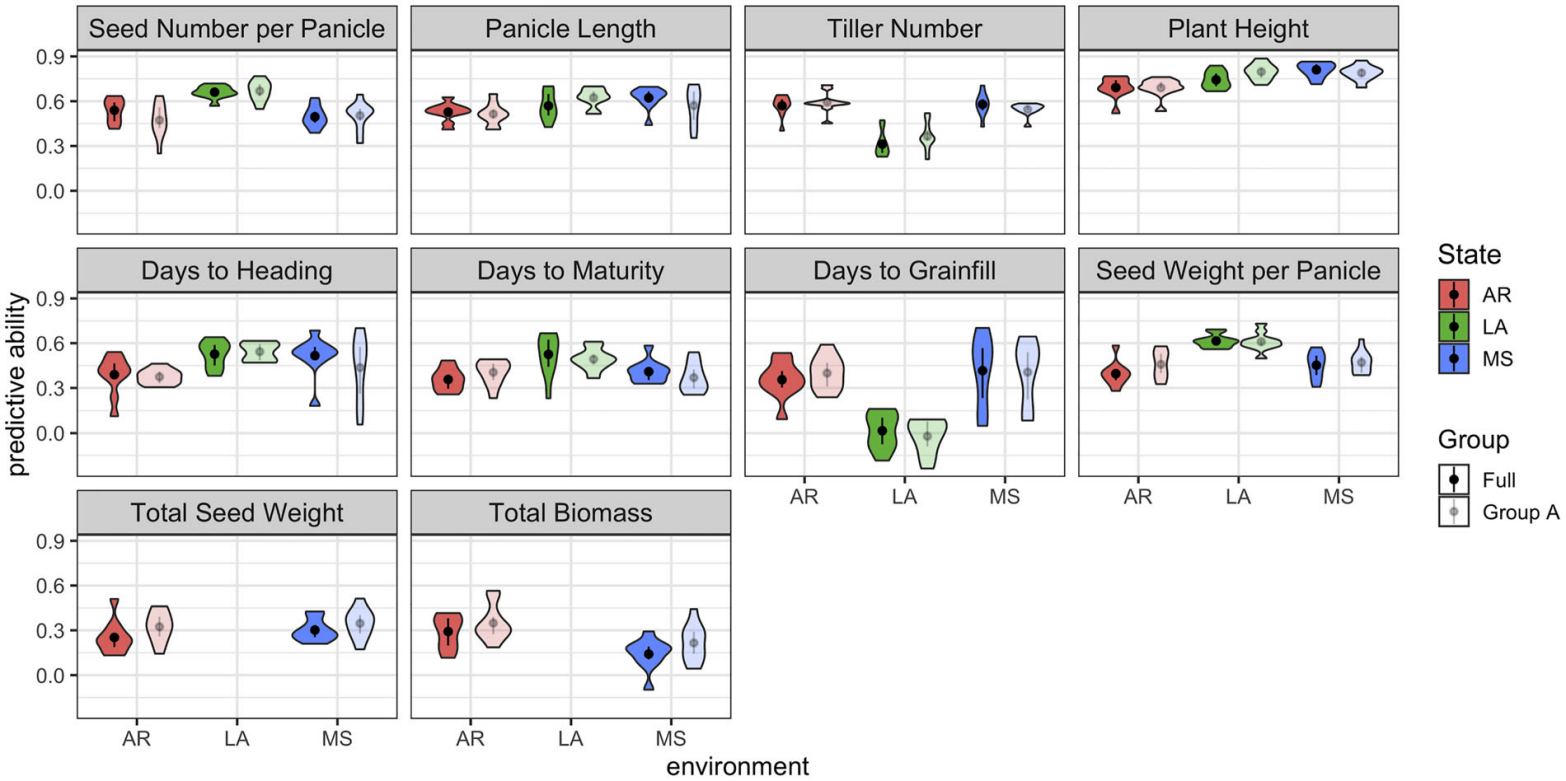
Single‐trait genomic prediction with genomic best linear unbiased prediction (GBLUP) within each environment in all rice genotypes versus solely within the Group A genotypes. The median of each instance of fivefold cross‐validation (20 iterations) is depicted as the dot at the center of the violin. The violins are omitted for states where a given phenotype was not measured.

The highest predictive abilities within all three states were for plant height, followed by panicle length and seed number per panicle (Figure 5; Figure S6). The predictive ability was measured using GBLUP, which is a generally high‐performing method for GP, consistently outperforming other methods, and is standard to use even for traits with differing heritabilities (Azodi et al., 2019; Kaler et al., 2022). These three high‐predictive ability traits also had the highest variance explained by genotype in relation to environment (Figure 4). Predictive abilities for total seed weight and total biomass were consistently the lowest in the two environments in which they were measured.

In this study, GP performed the same within Group A as it did for the entire population (Figure 5). GP was only conducted in Group A because it had a sufficient sample size for GP, whereas Group B on its own did not. Separating the population into three groups using *k*‐means clustering (*k* = 3) based on the scree plot (Figure S7) would have resulted in all groups being too small for testing of GP schemas (Figure S8). While only including more similar genotypes (e.g., Group A) could be hypothesized to result in higher predictive abilities, such an improvement may have been offset by the smaller sample size in the training set. In a realistic GS scenario, breeding goals would be for a particular product profile (Cobb et al., 2019; Ragot et al., 2018); generally, making “good by good” crosses between genotypes with desirable breeding values for that profile (Bernardo, 2008). The lack of predictive disadvantage when including all genotypes suggests that prediction models in the future could consider including diverse genotypes to increase the size of the model training set.

Spindel and Iwata (2018) discussed the benefit of including data that have been acquired over years when implementing GS in rice to build better GP/GS models over time. The historical genotypic and plant‐scale phenotypic data used in this study could be beneficial to augment future datasets being utilized for GS by breeding programs with interrelated populations in the Mississippi Delta, particularly for any genotypes related to modern elite varieties. The literature has shown that including historical data can be beneficial in cases where data are limited (for example, in winter wheat; Ballén‐Taborda et al., 2022) but can cause increased bias in other scenarios (for example, in dairy sheep; Granado‐Tajada & Ugarte, 2024). Relatedly, a limitation of this study is that GP was only conducted with data from 1 year. A key next step would be to conduct a training set optimization study within and across the southern US rice breeding programs to determine if including relevant or related historical varieties, or varieties across market classes, would be beneficial to prediction across multiple environments.

### 3.4 Multi‐trait GP

**FIGURE 6:**
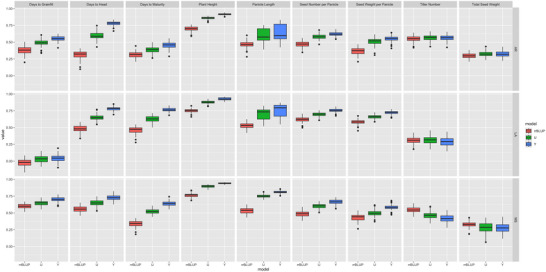
Mega‐scale linear mixed models (MegaLMM) predictive ability. MegaLMM predictive abilities are reported as the correlation coefficients for predicted versus observed *Y* (total genetic value) and *U* ​​(additive genetic value), as well as the predictive abilities from twofold cross‐validation of genomic best linear unbiased prediction (GBLUP) (via ridge regression best linear unbiased prediction [rrBLUP]) conducted with the same fold assignments. Color corresponds to the method used.

Multi‐trait GP using MegaLMM had higher predictive ability than single‐trait GP in the equivalent experimental structure for most traits and most environments (Figure 6; Table 1). The predictive advantage of multi‐trait prediction was strong for seed number per panicle, seed weight per panicle, panicle length, and plant height. These results suggest that further use of multi‐trait GP prediction in regional rice breeding could be advantageous. Two exceptions were that multi‐trait prediction performed the same as or worse than single‐trait GP for tiller number and total seed weight (Table 1). Tiller number had a large percentage of variance explained by each of the environment and the residual terms (Figure 4) alongside high coefficients of variation (Figure S9). Total seed weight was only measured in MS and AR and had 75% of variance explained by the residual (Figure S10). The highest‐magnitude phenotypic correlation for these traits was with each other (*r* = 0.540–0.613; Figure S5). The next highest magnitude of phenotypic correlation for total seed weight was with seed number per panicle and seed weight per panicle (*r* = 0.253–0.378), which had >40% variance explained by the residual (the highest for any trait measured in all three environments).

**TABLE 1:** Mean predictive ability from mega‐scale linear mixed models (MegaLMM) and rrBLUP.

State	Trait	*Y*	*U*	rrBLUP
MS	Days to grainfill	0.70	0.65	0.60
MS	Heading date	0.73	0.65	0.56
MS	Maturity date	0.64	0.52	0.34
MS	Plant height	0.94	0.90	0.76
MS	Panicle length	0.81	0.75	0.53
MS	Seed number per panicle	0.66	0.60	0.48
MS	Seed weight per panicle	0.58	0.50	0.43
MS	Tiller number	0.42	0.46	0.54
MS	Total seed weight	0.28	0.28	0.33
AR	Days to grainfill	0.55	0.49	0.38
AR	Heading date	0.78	0.59	0.31
AR	Maturity date	0.46	0.38	0.31
AR	Plant height	0.92	0.86	0.70
AR	Panicle length	0.65	0.61	0.47
AR	Seed number per panicle	0.62	0.58	0.47
AR	Seed weight per panicle	0.55	0.51	0.36
AR	Tiller number	0.56	0.57	0.55
AR	Total seed weight	0.32	0.32	0.30
LA	Days to grainfill	0.04	0.03	−0.03
LA	Heading date	0.78	0.65	0.48
LA	Maturity date	0.76	0.63	0.46
LA	Plant height	0.93	0.88	0.75
LA	Panicle length	0.76	0.70	0.53
LA	Seed number per panicle	0.75	0.70	0.62
LA	Seed weight per panicle	0.72	0.66	0.58
LA	Tiller number	0.29	0.31	0.31

*Note*: MegaLMM predictive abilities are reported as the correlation coefficients for predicted versus observed *Y* (total genetic value) and *U* (additive genetic value), as well as the predictive abilities from twofold cross‐validation of genomic best linear unbiased prediction (GBLUP) (via ridge regression best linear unbiased prediction [rrBLUP]) conducted with the same fold assignments.

Genetic and residual correlations and/or heritabilities (Runcie & Cheng, 2019) could have had a role in multi‐trait prediction not having outperformed single‐trait prediction for tiller number and total seed weight. The highest‐magnitude genetic correlation for tiller number was with seed number per panicle (*r *= −0.413 within MS, −0.290 within AR, and −0.401 between tiller number in MS and seed number per panicle in AR; Table S2). For total seed weight, no genetic correlations had a magnitude >0.3. In contrast, one or more genetic correlations with magnitudes ≥0.4 were generally observed for phenological traits (within and across environments) and between seed number per panicle and seed weight per panicle (within and across environments, except not between Arkansas and Louisiana). Panicle length and plant height did not have genetic correlations with magnitudes ≥0.4 with any trait (except *r* = 0.411 between plant height and days to heading in AR) but had a higher percentage of variance explained by *G*, and a lower percentage explained by the residual, than tiller number (Figure 4) and total seed weight (Figure S10)—particularly in the case of plant height.

Identification of higher‐heritability and consistently associated secondary traits for total seed weight (or grain yield, on a larger‐plot scale) would be helpful. For instance, Li et al. (2019) studied 7686 rice varieties released in China between 1978 and 2017 and found traits associated with yield for *japonica* inbreds and hybrids included panicle number per unit area and a long growth period, whereas several traits (including panicle length, filled grain number, 1000‐grain weight, and grain dimension traits) were found to be associated with yield for *indica* inbreds and hybrids. Partial phenotyping of primary and/or secondary traits in the test set could also be expected to have continued utility in multi‐trait prediction, as found by Shahi et al. (2022) for two physiological traits when predicting yield and four other primary traits in wheat, Bhatta et al. (2020) for agronomic traits when predicting yield—and those alongside malting traits when predicting grain protein content—in barley, and Persa et al. (2020) when using six associated agronomic or seed macronutrient traits to predict yield in soybean.

Previous studies have called for the use of multi‐trait prediction, including use of secondary traits as predictors for a focal trait (Arouisse et al., 2021; Robert et al., 2020; Xu et al., 2021), with previous applications in several crops including wheat (Gill et al., 2021, 2023; Montesinos‐López et al., 2021). Xu et al. (2021) described the importance of GP for rice breeding programs, especially integrating multiple traits to reduce costs, improve model accuracy, and select for multiple traits simultaneously. Additional key considerations for GP/GS in rice include improving selection accuracy, incorporating G × E interactions into the prediction, and being aware of the impacts of population size and structure (Xu et al., 2021). A further practical value of multi‐trait prediction is the potential to predict unmeasured traits and reduce costs of phenotyping. If only a subset of the traits (or the less costly traits) of interest are measured, multi‐trait prediction could be used to fill in the missing (or more costly) traits. For example, seed weight per panicle and seed number per panicle having been so closely phenotypically correlated (*r* = 0.919 using across‐environment averages) and genetically correlated (>0.6 within environments; Table S2) suggests that the former, which is typically easier to measure, could be beneficial in multi‐trait prediction of the latter. Another practical application could be predicting yield traits earlier in the season using yield‐component traits that have already been phenotyped, or predicting into untested environments in the southern US. For instance, such methods could be useful if yield is lost due to a late‐season weather event, but other associated traits were measured earlier in the season.

Advancements in incorporating phenotypes and environmental conditions as covariates have improved statistical prediction. Liang et al. (2023) proposed a different way of incorporating correlated phenotypes using machine learning approaches, including multi‐target regressors to extract and include phenotypic data in predictions. Another approach for incorporating phenotypes into prediction has been presented for the impact of high night temperatures on rice grain quality (Bi et al., 2024). That study used the measurements of multiple metabolites and a joint analysis of these factors as covariates in predicting other related metabolites. Environmental covariates are another approach for improving predictions, particularly if data from a large number of sites and trials are available (Hu et al., 2024). Another avenue to combine phenotypic data, environmental information, and management records to improve prediction is integrating GP and crop growth modeling using the simulation of intermediate traits. This practice has been well‐established for yield and other agronomic traits in maize (Cooper et al., 2016; Diepenbrock et al., 2021; Messina et al., 2018; Technow et al., 2015) and has had some success in rice (Onogi et al., 2016; Yang et al., 2022). Incorporating grain quality traits (e.g., nutritional composition, or grain chalkiness under reproductive‐stage high‐temperature stress; Shi et al., 2017) would be a major next step.

## 4 CONCLUSION

This project began as a multistate and multi‐institution cooperative project to integrate genetic and phenotypic data for the purpose of understanding traits relevant to US southern rice breeding programs. The population has been described herein, and the data have been used to test single‐ and multi‐trait prediction scenarios, which was feasible due to the genetic diversity represented in these genotypes and detailed phenotyping of phenological, architectural, and yield‐component traits across environments. Because this population was genotyped with the 550 SNP panel, the genetic data were better suited for GP than a genome‐wide association study. A future direction with this population could be to further an original goal of RiceCAP: linking genotype to phenotype to better understand the genetic underpinnings of agronomic traits. Those genome‐wide association study results could then be incorporated into GS (e.g., with peak markers for major‐effect genes incorporated as fixed effects) to further the breeding of US rice (as described in Bhandari et al. [2019, Spindel et al. [2015]], and Xu et al. [2021]).

In conclusion, population structure analyses showed that, among the 429 genotypes grown and phenotyped, there was a genetic distinction between the *japonica*‐originating genotypes and *indica*‐originating genotypes in the PC space (Figure 1A; Table S1). Additionally, the grouping of vectors within traits (rather than within environments) in Figure 2A showed the importance of genotype. G × E analyses suggested the importance of environment‐aware predictions in addition to classical GP (Figure 4). Multi‐trait prediction using MegaLMM generally conferred a predictive advantage (Figure 6). The results from this large, diverse set of rice genotypes could inform future GP/GS implementations for continued genetic gain in southern US rice.

## AUTHOR CONTRIBUTIONS

**Mary‐Francis LaPorte**: Conceptualization; formal analysis; funding acquisition; methodology; visualization; writing—original draft; writing—review and editing. **Haixiao Hu**: Methodology; writing—review and editing. **Walter Solomon**: Data curation. **James H. Oard**: Data curation. **Steven J. Knapp**: Methodology; writing—review and editing. **Daniel E. Runcie**: Methodology; writing—review and editing; **Jeremy D. Edwards**: Conceptualization; data curation; writing—review and editing. **Anna McClung**: Conceptualization; data curation; writing—review and editing. **Christine H. Diepenbrock**: Conceptualization; funding acquisition; methodology; supervision; writing—original draft; writing—review and editing.

## CONFLICT OF INTEREST STATEMENT

The authors declare no conflicts of interest.

## Supporting information



Supplemental File 1: Supplemental Figures S1‐S10 include: (S1) the Köppen‐Geiger map showing the location of the three field sites in Arkansas (AR), Mississippi (MS) and Louisiana (LA); (S2) ADMIXTURE software cross‐validation error, (S3) heatmap of the kinship matrix, (S4) two‐state principal component (PC) analysis, (S5) correlogram of phenotypic data, (S6) observed vs. predicted values for genomic prediction, (S7) scree plot for the genotypic PCA analysis, (S8) PCA grouped by k = 3, (S9) the coefficient of variation for the traits in each environment, and (S10) two‐state percent variance explained. This file also includes two supplemental tables. Supplemental Table 1 includes mean MegaLMM values, and Supplemental Table 2 includes genetic correlations.
Supplemental File 2: Genotype annotations
Supplemental File 3: Eigenvalues for genotypic PCA (*eigenvalues_RiceCAP_AMP_083023.txt*)
Supplemental File 4: Scatterplots of measured phenotypes vs. model predictions for multi‐trait genomic prediction conducted via MegaLMM.

Supplemental Material

Supplemental Material

Supplemental Material

Supplemental Material

Supplemental Material

## Data Availability

All scripts used in this study will be made available as additional files within the Supporting Information during the review period and through a public GitHub repository at the time of publication at https://github.com/laporpe/RiceCAP. Accession annotations are included in the supplemental materials for this manuscript, which will be hosted on the journal website. The genotypic data and phenotypic data are hosted on the Dryad data repository at https://doi.org/10.5061/dryad.j9kd51ctd.
