# Supplementary material for: Multi‐environment evaluation and genomic prediction of agronomic traits in the southern US rice genepool
Source: Plant Genome. 2026 Mar 30;19(2):e70222. doi: 10.1002/tpg2.70222 (PMC13034092; doi:10.1002/tpg2.70222)
Supplement: Supplementary file 1 — Supplemental File 1: Supplemental Figures S1‐S10 include: (S1) the Köppen‐Geiger map showing the location of the three field sites in Arkansas (AR), Mississippi (MS) and Louisiana (LA); (S2) ADMIXTURE software cross‐validation error, (S3) heatmap of the kinship matrix, (S4) two‐state principal component (PC) analysis, (S5) correlogram of phenotypic data, (S6) observed vs. predicted values for genomic prediction, (S7) scree plot for the genotypic PCA analysis, (S8) PCA grouped by k = 3, (S9) the coefficient of variation for the traits in each environment, and (S10) two‐state percent variance explained. This file also includes two supplemental tables. Supplemental Table 1 includes mean MegaLMM values, and Supplemental Table 2 includes genetic correlations. Supplemental File 2: Genotype annotations Supplemental File 3: Eigenvalues for genotypic PCA (eigenvalues_RiceCAP_AMP_083023.txt) Supplemental File 4: Scatterplots of measured phenotypes vs. model predictions for multi‐trait genomic prediction conducted via MegaLMM. [file TPG2-19-e70222-s005.docx]

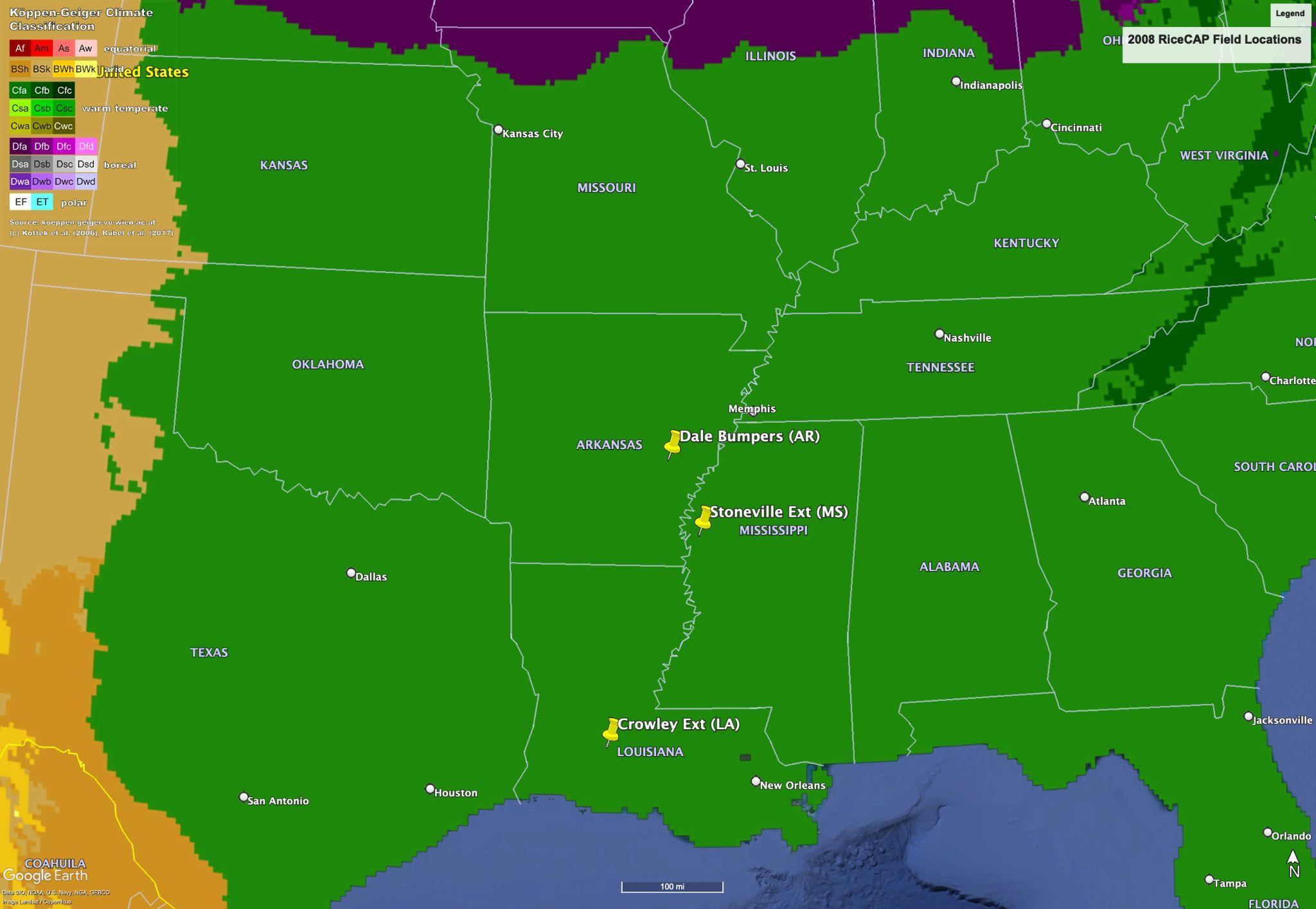


Supplemental Figure S1: Supplemental Figure qGIS: Köppen-Geiger map showing the locations of the three field sites in Arkansas (AR), Mississippi (MS) and Louisiana (LA). These field sites are in/near the municipalities of Stuttgart, AR (latitude: 34.5°), Stoneville, MS (33.4°), and Crowley, LA (30.4°). There is a 546 km distance between Crowley, LA and Stuttgart, AR. All three sites were in the Cfa environment, which is a temperate humid subtropical environment characterized by a hot summer and no dry season.


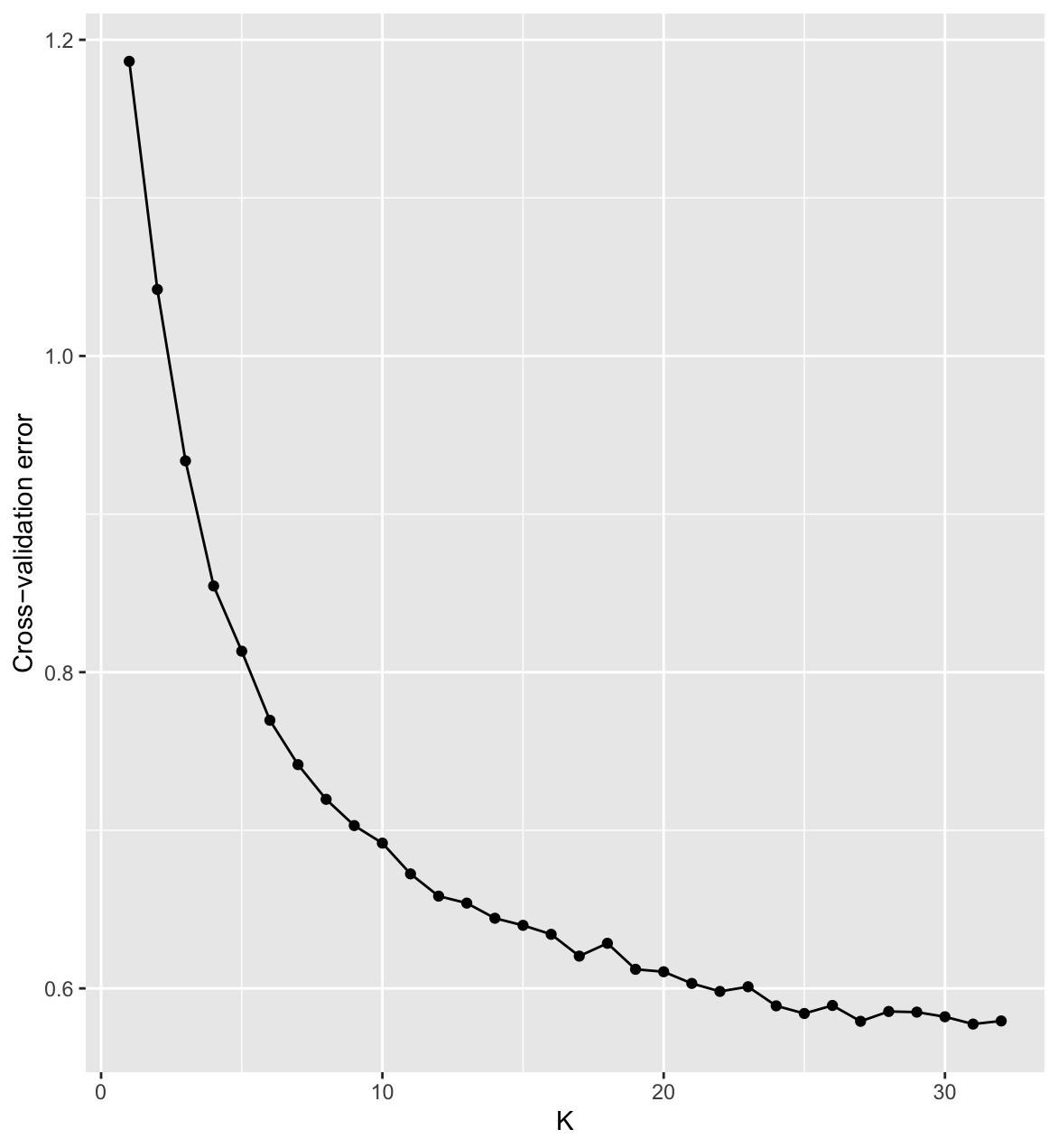


Supplemental Figure S2: Cross-validation error for each value of K (K = 1-32) using the ADMIXTURE software for population structure analysis.

**
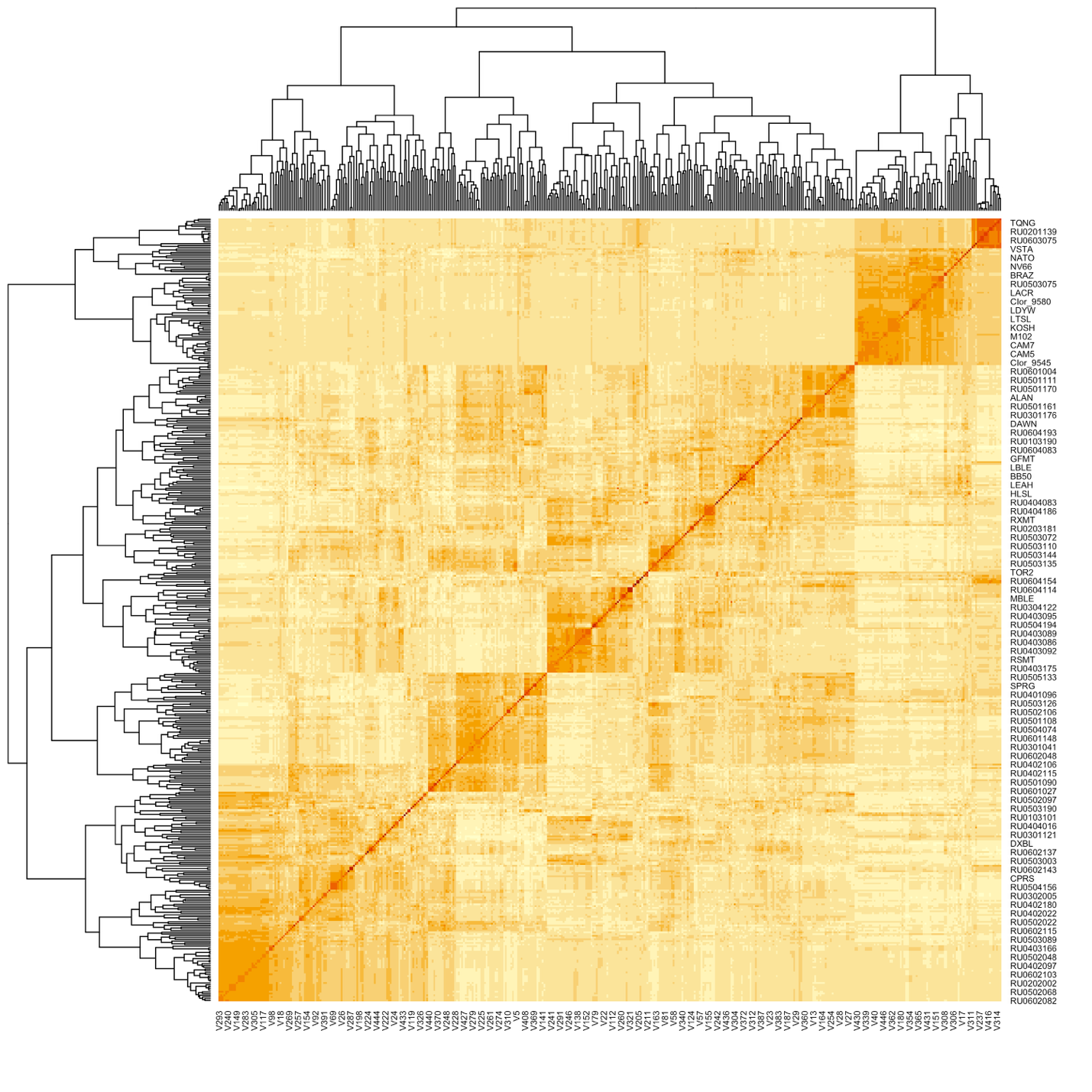
**

Supplemental Figure S3: A heatmap of the kinship matrix of the genotypes included in this study. Red depicts a higher correlation between genotypes.


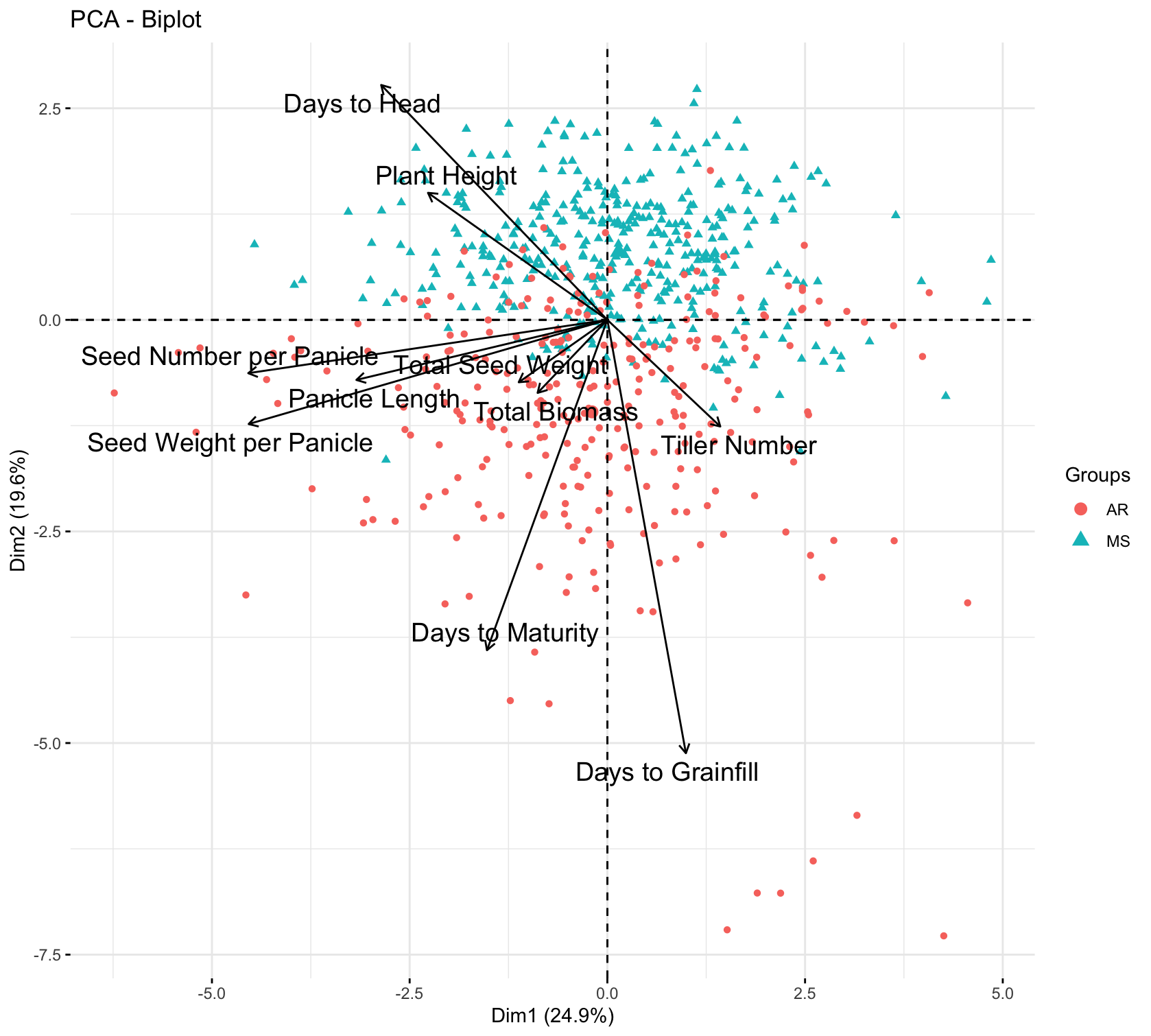


Supplemental Figure S4: Phenotypic PCA of traits including total seed weight and total biomass in the two environments in which they were measured (AR= Arkansas, MS = Mississippi).


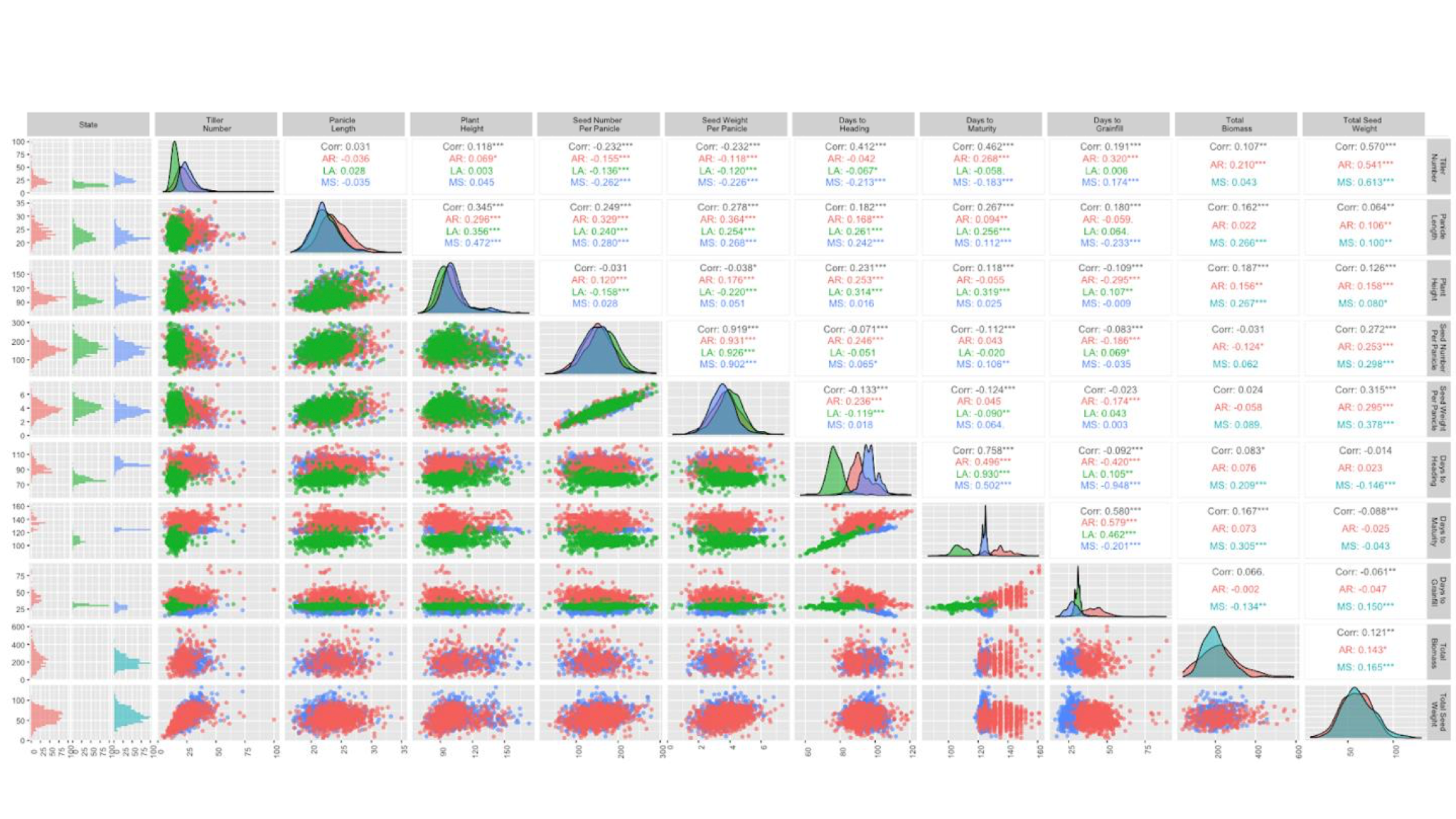


Supplemental Figure S5. Correlogram for phenotypes measured in each environment. The colors represent the state in which phenotypes were measured (AR: Arkansas in red; LA: Louisiana in green; MS: Mississippi in blue). For most traits and most environments 429 lines were measured (with the exception of Total Biomass, which was only measured for 157 lines in Arkansas and 198 lines in Mississippi).


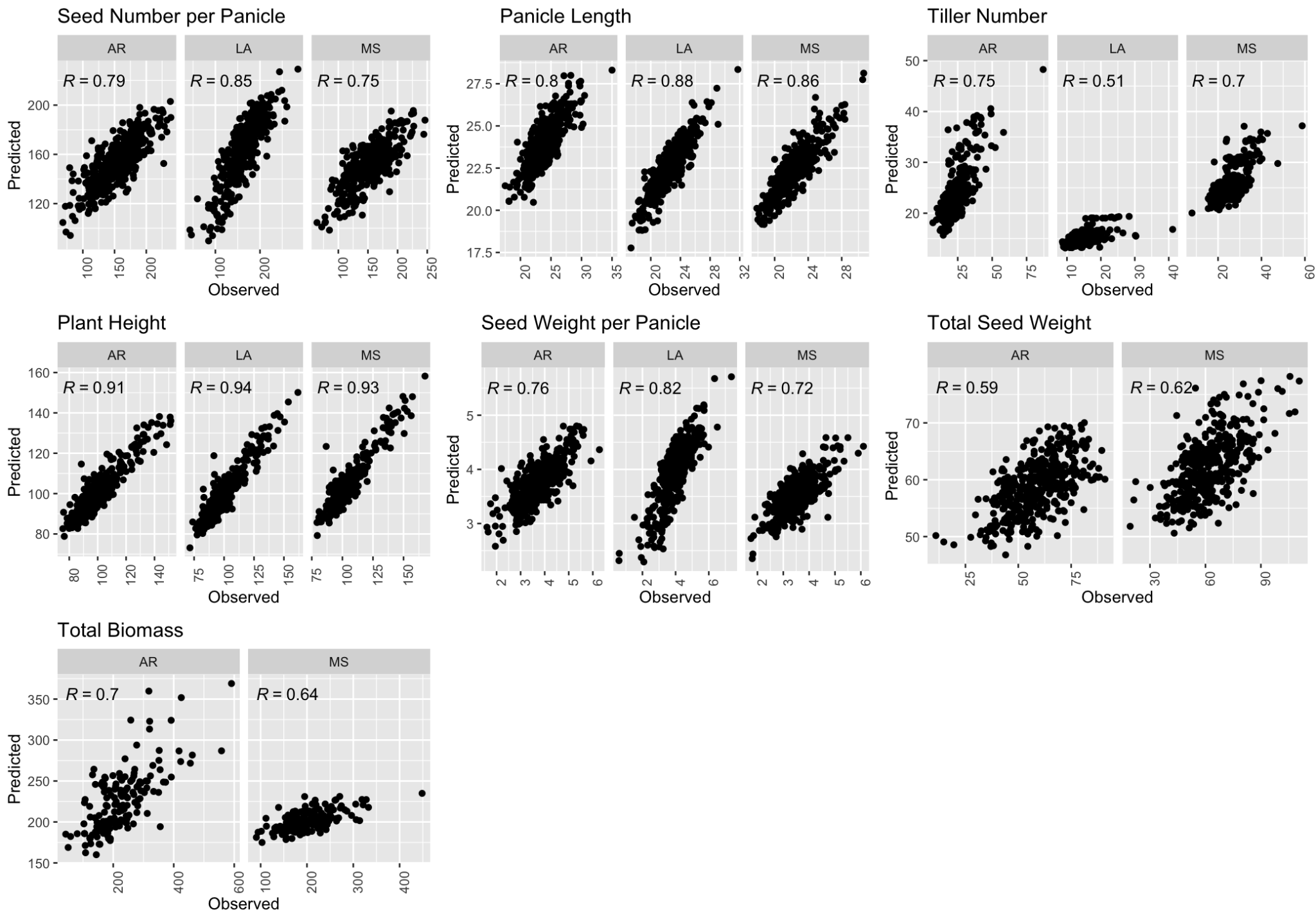


Supplemental Figure S6: Observed vs. Expected GBLUP: Observed vs predicted values for Genomic Prediction conducted via GBLUP. Correlation coefficients are reported for each trait and environment.


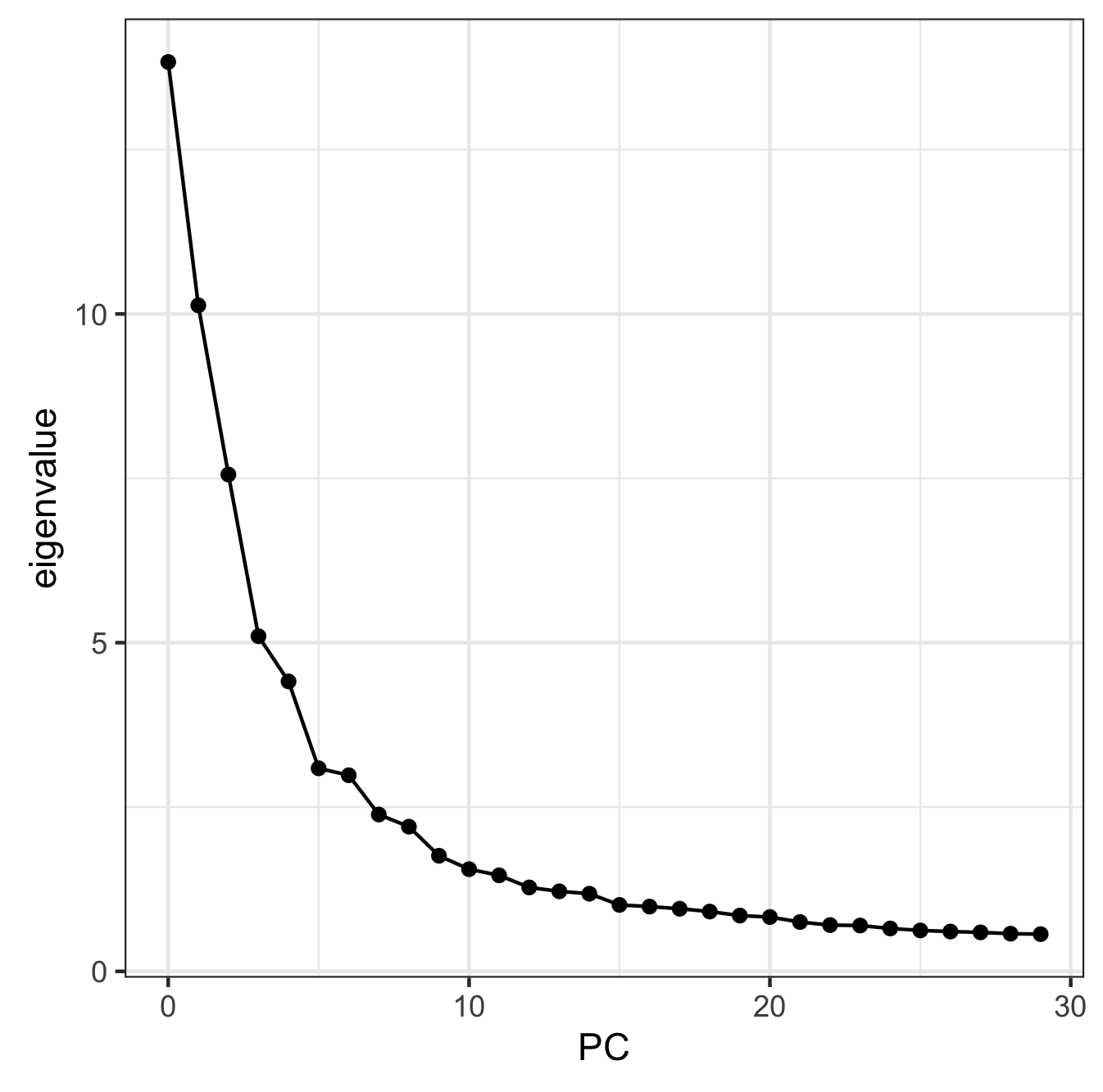


Supplemental Figure S7. Scree-plot for genotypic PCA depicts eigenvalue for each principal component (PC).


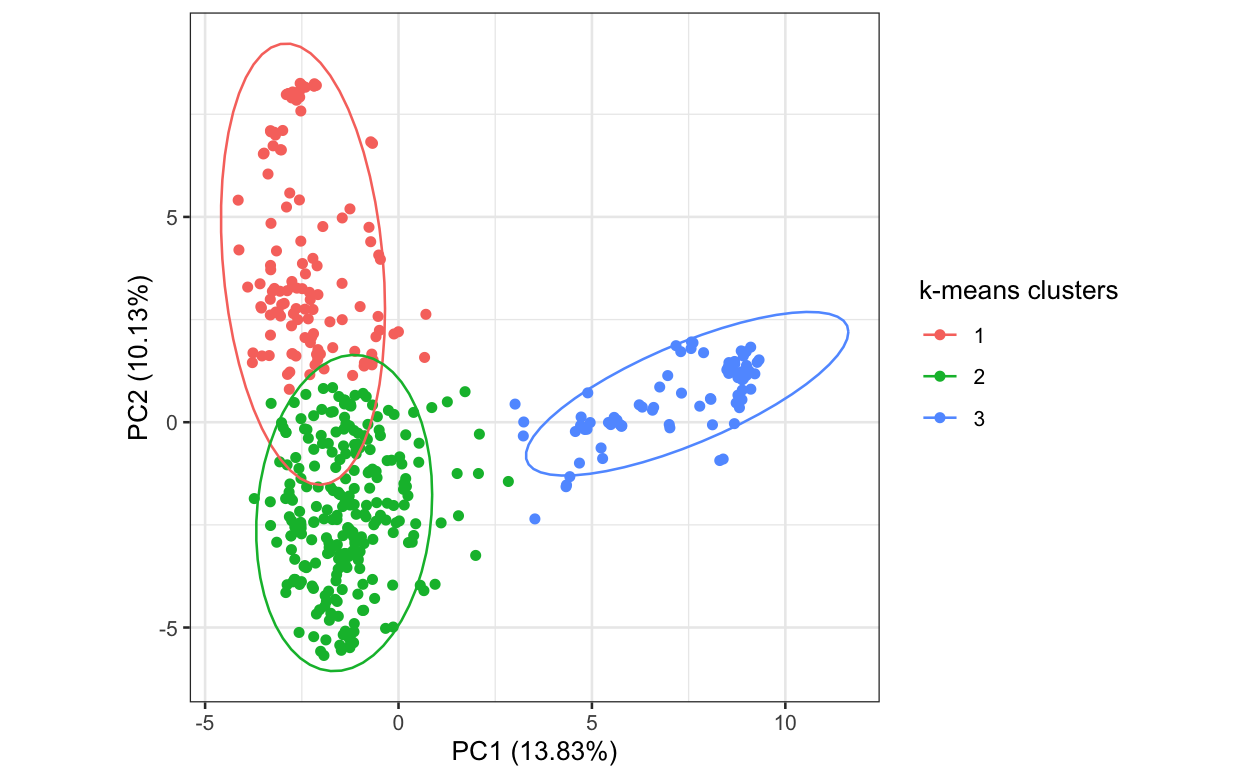


Supplemental Figure S8. Principal component (PC) analysis of genotypic data showing PC1 vs PC2. PCA plot color-coded to represent the three groups identified through k-means clustering. The percent variance explained by each PC is reported in parentheses in the respective axis label.


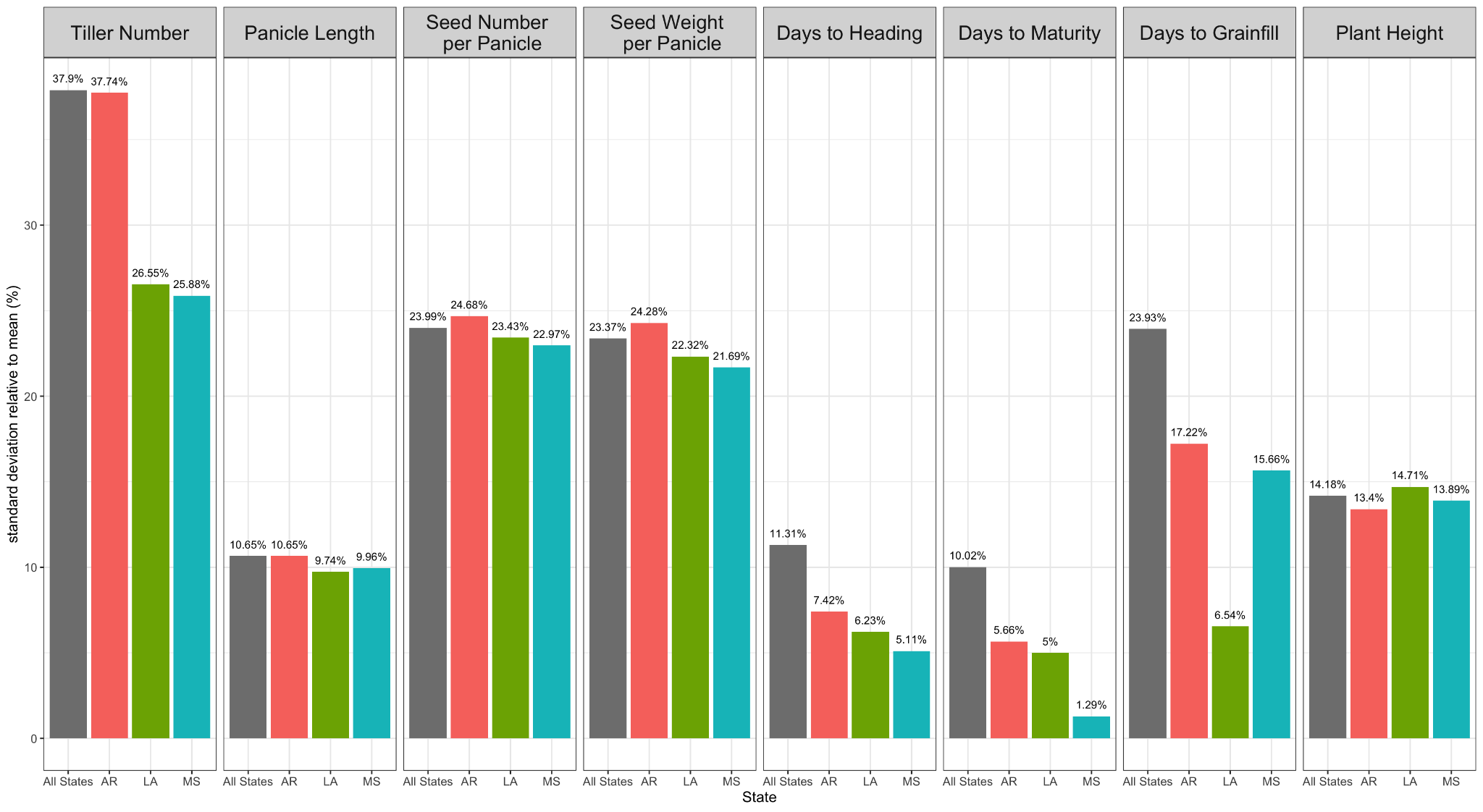


Supplemental Figure S9. The coefficient of variation ((standard deviation / mean) * 100, expressed as a percent) for the traits in each environment. The coefficient of variation for a given trait across all environments is represented by the leftmost gray bar, and the state-specific bars are color-coded AR: Arkansas (red); LA: Louisiana (green); or MS: Mississippi (blue). For each plot, the coefficient of variation in all environments is plotted next to the coefficient of variation in each environment.


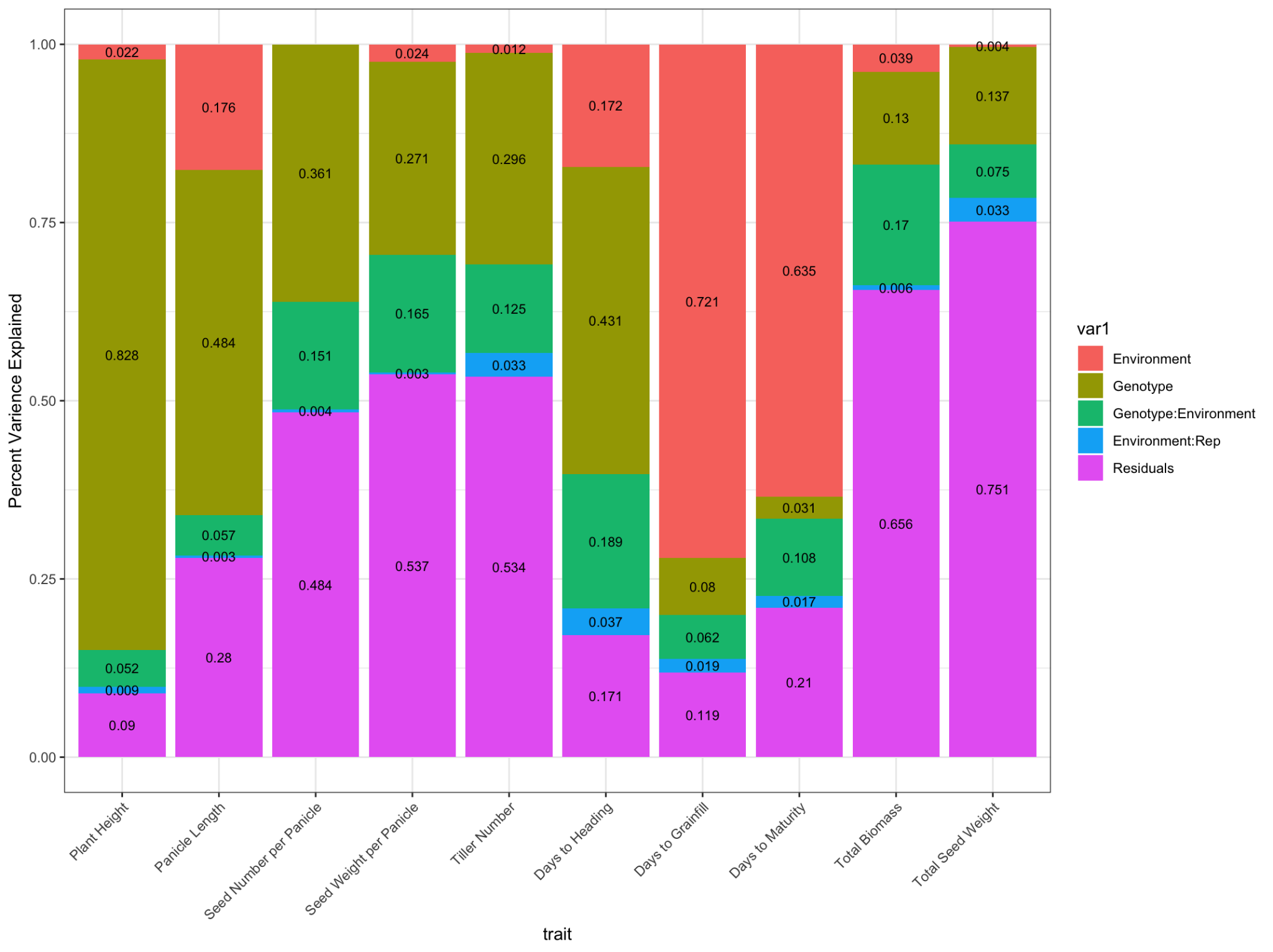


Supplemental Figure S10: Percent variance explained in the two states in which total biomass and total seed weight were measured (Arkansas and Mississippi).

Supplemental Table 1: Mean MegaLMM values. MS = Mississippi, LA = Louisiana, AR = Arkansas. Y = total genetic value; U = estimated additive genetic value.

| **State** | **trait** | **Y** | **U** | **rrBLUP** |
| --- | --- | --- | --- | --- |
| MS | 1_DaysGrainfill | 0.7 | 0.65 | 0.6 |
| MS | 1_DaysHEAD | 0.73 | 0.65 | 0.56 |
| MS | 1_DaysMATURITY | 0.64 | 0.52 | 0.34 |
| MS | 1_MEANHT | 0.94 | 0.9 | 0.76 |
| MS | 1_PANLENGTHPLANTMEAN | 0.81 | 0.75 | 0.53 |
| MS | 1_SEEDNUMperPANMEAN | 0.66 | 0.6 | 0.48 |
| MS | 1_SEEDSAMPLEwtPANmean | 0.58 | 0.5 | 0.43 |
| MS | 1_TILLNUMMEAN | 0.42 | 0.46 | 0.54 |
| MS | 1_TOTSEEDWTmean | 0.28 | 0.28 | 0.33 |
| AR | 2_DaysGrainfill | 0.55 | 0.49 | 0.38 |
| AR | 2_DaysHEAD | 0.78 | 0.59 | 0.31 |
| AR | 2_DaysMATURITY | 0.46 | 0.38 | 0.31 |
| AR | 2_MEANHT | 0.92 | 0.86 | 0.7 |
| AR | 2_PANLENGTHPLANTMEAN | 0.65 | 0.61 | 0.47 |
| AR | 2_SEEDNUMperPANMEAN | 0.62 | 0.58 | 0.47 |
| AR | 2_SEEDSAMPLEwtPANmean | 0.55 | 0.51 | 0.36 |
| AR | 2_TILLNUMMEAN | 0.56 | 0.57 | 0.55 |
| AR | 2_TOTSEEDWTmean | 0.32 | 0.32 | 0.3 |
| LA | 3_DaysGrainfill | 0.04 | 0.03 | -0.03 |
| LA | 3_DaysHEAD | 0.78 | 0.65 | 0.48 |
| LA | 3_DaysMATURITY | 0.76 | 0.63 | 0.46 |
| LA | 3_MEANHT | 0.93 | 0.88 | 0.75 |
| LA | 3_PANLENGTHPLANTMEAN | 0.76 | 0.7 | 0.53 |
| LA | 3_SEEDNUMperPANMEAN | 0.75 | 0.7 | 0.62 |
| LA | 3_SEEDSAMPLEwtPANmean | 0.72 | 0.66 | 0.58 |
| LA | 3_TILLNUMMEAN | 0.29 | 0.31 | 0.31 |

Supplemental Table 2:

Genetic correlations: *Genetic_Correlation_Matrix.xlsx*

Supplemental File 2: Pedigree (or entry name) abbreviated with the annotation. The empirical annotation (k-means clustering of the genotypic PCA) and the manual annotations (state of origin, “State”) are reported when available. This file also includes the pedigree for all lines in which it is available, as well as the primary parents in the cross when available.

*Geno_annotations_supplement.xlsx*

Supplemental File 3: Eigenvalues for genotypic PCA (*eigenvalues_RiceCAP_AMP_083023.txt*)

Supplemental File 4: Observed vs. expected values calculated using MegaLMM. The correlation coefficient between the observed and expected is annotated for each trait and environment.

*MegaLMM-plots-forSupp.pptx*
