# Supplementary material for: Multi‐environment evaluation and genomic prediction of agronomic traits in the southern US rice genepool
Source: Plant Genome. 2026 Mar 30;19(2):e70222. doi: 10.1002/tpg2.70222 (PMC13034092; doi:10.1002/tpg2.70222)

Mississippi MegaLMM Scenario 1: Observed (model predictions) vs Expected (measured phenotypes)

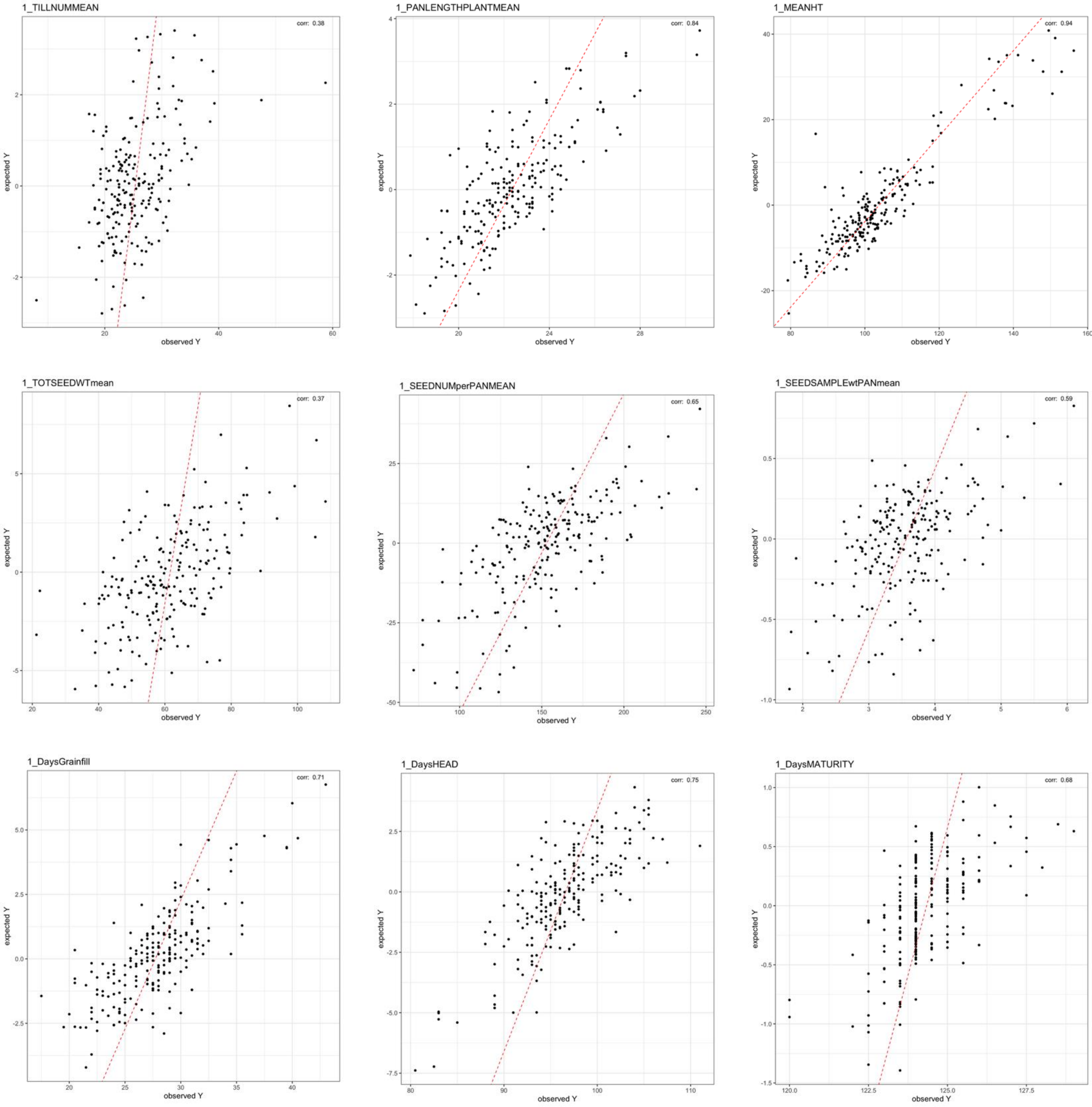

Arkansas MegaLMM Scenario 1: Observed (model predictions) vs Expected (measured phenotypes)

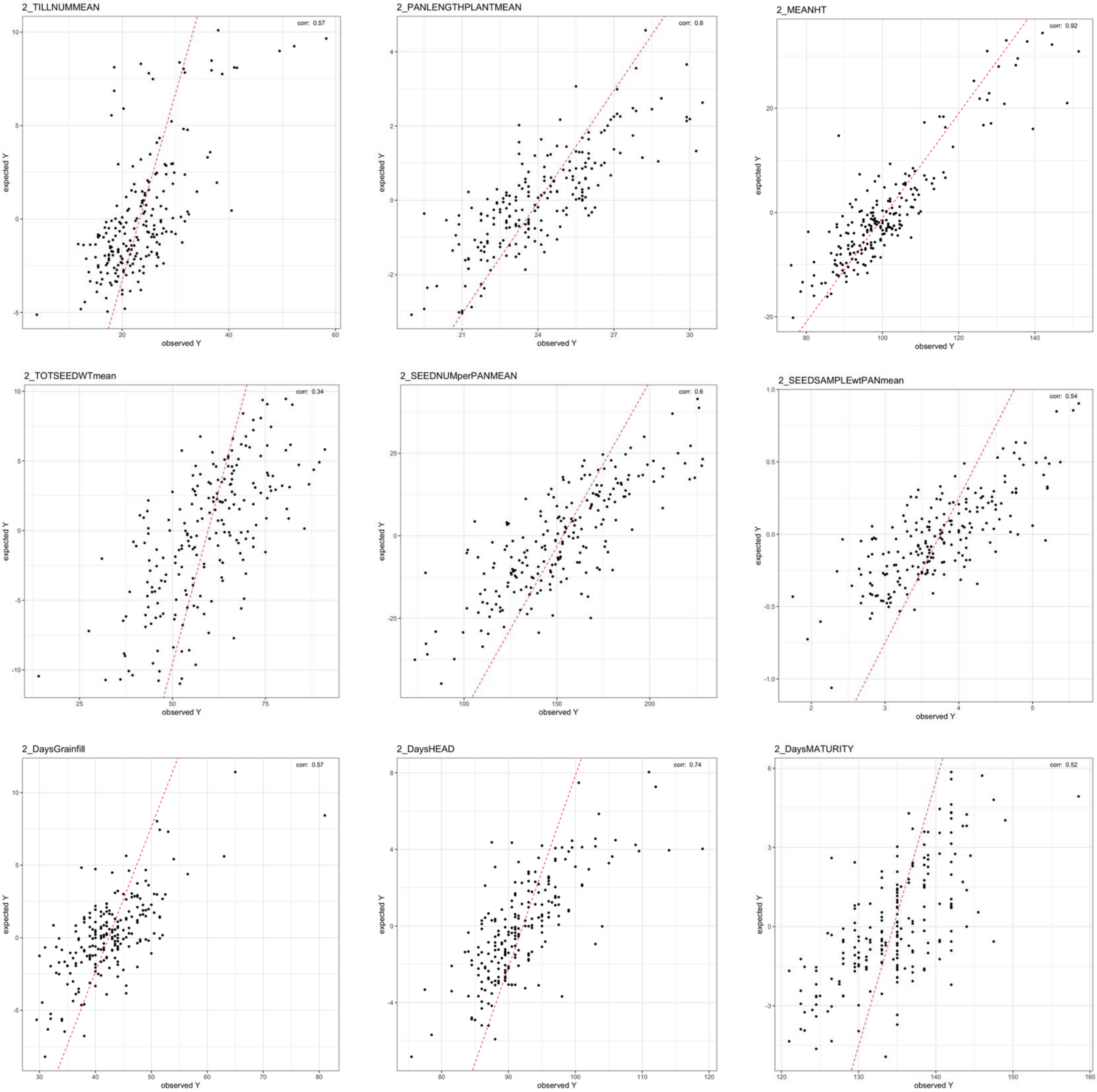

Louisiana MegaLMM Scenario 1: Observed (model predictions) vs Expected (measured phenotypes)

3_TILLNUMMEAN

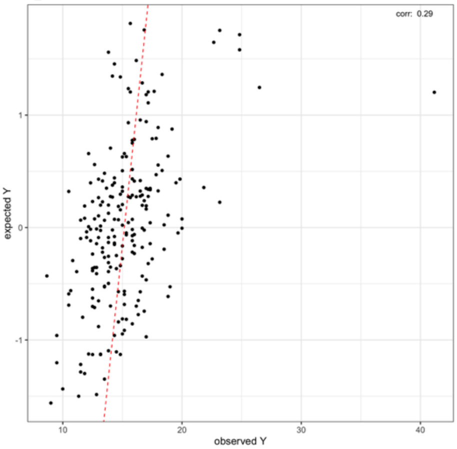

3_PANLENGTHPLANTMEAN

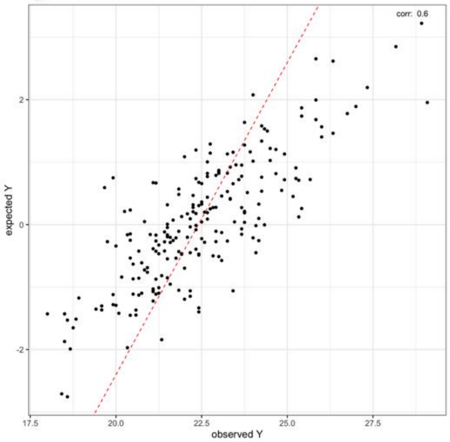

3_MEANHT

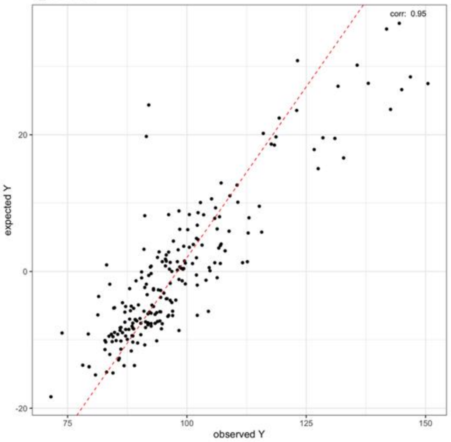

3_SEEDNUMperPANMEAN

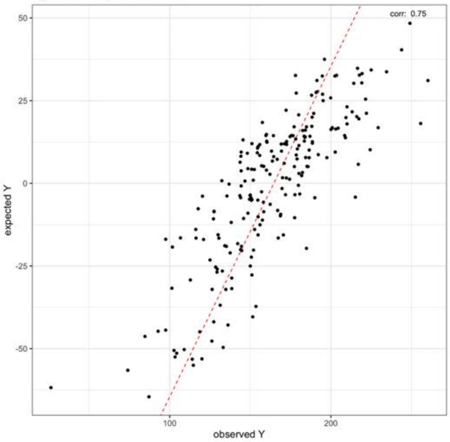

3_SEEDSAMPLEwtPANmean

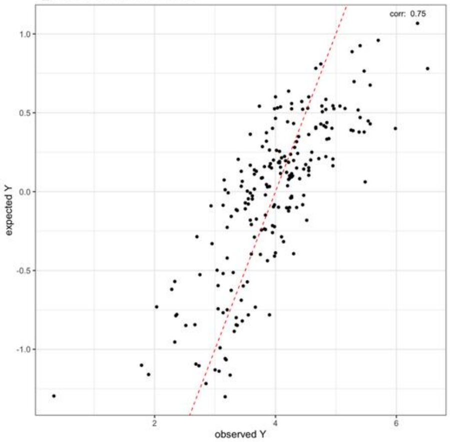

3_DaysGrainfill

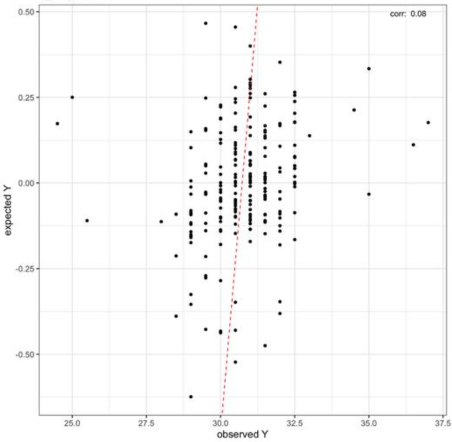

3_DaysHEAD

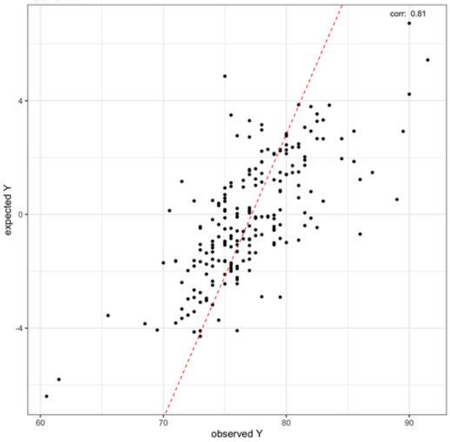

3_DaysMATURITY

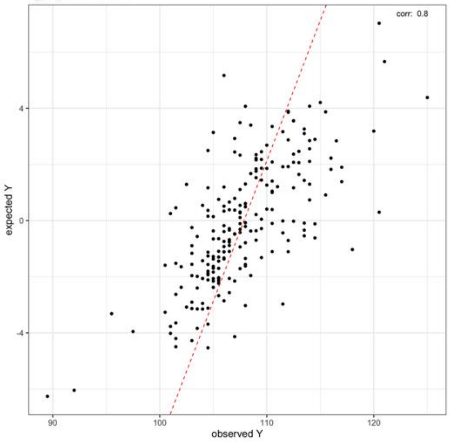

Supplement: Supplementary file 5 — Supplemental Material [file TPG2-19-e70222-s001.pdf]
